# Dissection of Microglial Heterogeneity and Identification of Pain‐Related Subpopulations After Traumatic Brain Injury

**DOI:** 10.1155/prm/2444554

**Published:** 2026-09-08

**Authors:** Fu Zhao, Jingru Shi, Yimin Zhang, Luxi Cao

**Affiliations:** ^1^ School of Traditional Chinese Medicine, Jinan University, Guangzhou 510632, China, jnu.edu.cn; ^2^ Department of Plastic and Reconstructive Surgery, Shanghai Ninth People’s Hospital, Shanghai Jiao Tong University School of Medicine, Shanghai, China, shsmu.edu.cn

**Keywords:** microglia, neuroinflammation, pain, single-cell transcriptomics, traumatic brain injury

## Abstract

**Background:**

Pain following traumatic brain injury (TBI) is a major challenge in clinical management. Neuroinflammatory responses, particularly those involving microglia, are increasingly implicated in the synaptic and circuit‐level remodeling associated with post‐TBI pain. However, the conventional M1/M2 framework does not adequately capture the temporal and functional diversity of microglial states after injury. This study aimed to characterize microglial heterogeneity and identify specific microglial subpopulations potentially associated with post‐TBI pain–related neural remodeling.

**Methods:**

We reanalyzed the publicly available single‐cell RNA sequencing dataset GSE226207, which comprised intact cortical samples and cortical samples collected at 3 and 5 days after cortical stab‐wound injury from control and inhibitor‐treated mice. The inhibitor‐treated groups received combined pharmacological inhibition of the CXCR3 and TLR1/2 pathways. Seurat was used for data processing, clustering, cell‐type annotation, and microglial subclustering. AUCell was applied to evaluate inflammatory‐, repair‐, and pain‐relevant gene signatures. CytoTRACE and Slingshot were used to assess relative transcriptional complexity and infer state‐transition trajectories. CellChat analysis of pooled cells from all groups was performed to predict intercellular communication, and SCENIC was used to characterize subpopulation‐associated regulon activity.

**Results:**

A total of 66,310 cells were retained, including 7648 microglia that were resolved into eight transcriptionally distinct subpopulations. Among them, the *L* *a* *r* *s*2^+^ microglial subpopulation showed lower representation in the 3 days postinjury (dpi) groups and greater representation in the 5 dpi groups. This subpopulation exhibited relatively high TGF‐β– and MAPK‐associated signature scores and was enriched in biological processes related to synaptic organization, axonogenesis, dendrite development, and protein synthesis. Slingshot positioned *L* *a* *r* *s*2^+^ microglia at the distal end of one inferred state‐transition lineage. CellChat analysis predicted prominent neuron‐to‐microglia communication through the CX3CL1–CX3CR1 ligand–receptor pair, with *L* *a* *r* *s*2^+^ microglia predominantly positioned as signal receivers. SCENIC further identified relatively high activity of regulatory module M1 in *L* *a* *r* *s*2^+^ microglia, with *Thra* among the highly ranked regulators.

**Conclusion:**

This study identifies the *L* *a* *r* *s*2^+^ microglial subpopulation as a temporally dynamic and transcriptionally specialized component of the postinjury microglial landscape. The convergence of pain‐relevant signaling signatures, neural remodeling programs, inferred state‐transition characteristics, predicted neuron–microglia communication involving the CX3CL1–CX3CR1 pair, and *Thra*‐associated regulon activity supports a potential role for *L* *a* *r* *s*2^+^ microglia in posttraumatic neuroimmune and synaptic remodeling. These findings provide a cellular and molecular framework for future investigation of microglia‐targeted strategies for post‐TBI pain.

## 1. Introduction

Traumatic brain injury (TBI) is defined as an alteration in brain function or other evidence of brain pathology caused by an external force and remains a major cause of death and long‐term neurological disability worldwide [1–3]. Pain is among the most common and persistent complications after TBI and may present as headache, neuropathic pain, musculoskeletal pain, or mixed pain syndromes [4, 5]. For some patients, pain persists well beyond the acute injury phase and substantially interferes with functional recovery, sleep, emotional health, and quality of life [4, 6]. Despite its clinical importance, post‐TBI pain remains difficult to manage because currently available treatments often provide incomplete or inconsistent relief, while mechanism‐based interventions targeting neuroimmune and neural plasticity processes remain limited [4, 6, 7]. A clearer understanding of the neuroimmune processes underlying posttraumatic pain is therefore needed to support the development of more precise interventions.

Beyond the initial mechanical insult, TBI initiates a secondary injury cascade involving blood–brain barrier disruption, excitotoxicity, oxidative stress, neuroinflammation, synaptic dysfunction, and progressive neuronal damage [8, 9]. Persistent neuroinflammatory signaling may alter neuronal excitability, impair endogenous pain‐modulatory mechanisms, and promote maladaptive plasticity within pain‐related neural networks [4, 9, 10]. Microglia are among the earliest cellular responders to brain injury. Following TBI, they accumulate around injured regions and participate in debris clearance, immune regulation, and tissue repair [11, 12]. When microglial responses become prolonged or dysregulated, they may sustain neuroimmune activation and influence synaptic remodeling, thereby contributing to pain‐related neural plasticity and the persistence of posttraumatic pain [13–15]. Although astrocytes and oligodendrocytes also shape the inflammatory and reparative environment after TBI [16, 17], microglia occupy a particularly important position at the interface of injury sensing, neuroimmune regulation, and synaptic remodeling. This makes microglial heterogeneity especially relevant to understanding the cellular mechanisms associated with post‐TBI pain.

Historically, microglial responses have often been interpreted through the M1‐/M2‐like polarization framework. Although this framework provides a useful conceptual basis for distinguishing broadly inflammatory and reparative phenotypes, it does not adequately capture the heterogeneity, plasticity, and context dependence of microglial states in vivo [18]. Advances in single‐cell RNA sequencing (scRNA‐seq) have revealed disease‐associated and injury‐responsive microglial states with distinct inflammatory, proliferative, metabolic, and reparative transcriptional features [19–21]. Collectively, these studies support a view of microglial activation as a dynamic and multidimensional process rather than a fixed binary program. However, the specific microglial states associated with pain‐related neuroimmune responses and synaptic remodeling after TBI remain poorly defined.

Microglial responses evolve dynamically across the course of secondary injury. Programs associated with acute inflammation may differ from those linked to subsequent repair and persistent neural plasticity, with corresponding changes in the abundance and transcriptional features of individual microglial states [22, 23]. Resolving these temporal dynamics is particularly relevant to posttraumatic pain, as mechanisms underlying early nociceptive signaling may differ from those that sustain maladaptive plasticity [4, 10]. However, the temporal relationships between microglial heterogeneity, pain‐associated transcriptional programs, neuron–microglia communication, and regulatory networks after TBI remain poorly defined at single‐cell resolution.

Against this background, we hypothesized that early traumatic cortical injury is accompanied by temporally distinct microglial states, some of which exhibit transcriptional programs related to inflammation, synaptic remodeling, and pain. We therefore reanalyzed a publicly available single‐cell transcriptomic dataset comprising intact cortex and early postinjury conditions [16]. By integrating microglial subclustering, functional signature scoring, trajectory inference, intercellular communication analysis, and transcriptional regulatory network analysis, we aimed to identify microglial states and signaling programs associated with post‐TBI pain–related neuroimmune and synaptic remodeling. This analysis delineates microglial heterogeneity after TBI at single‐cell resolution and prioritizes signaling pathways for subsequent mechanistic validation.

## 2. Methods

### 2.1. Source of scRNA‐seq Data

The scRNA‐seq data analyzed in this study were obtained from the Gene Expression Omnibus database under Accession No. GSE226207. The dataset was generated from 8‐ to 12‐week‐old male C57BL/6J mice subjected to cortical stab‐wound injury. Tissue samples were collected from the lesioned somatosensory cortical gray matter at 3 and 5 days postinjury, together with anatomically matched intact cortical samples. In the inhibitor‐treated groups, mice received combined pharmacological inhibition of the CXCR3 and TLR1/2 pathways through coadministration of the CXCR3 antagonist NBI 74330 and the TLR1/2 inhibitor CU CPT 22.

The dataset comprised 15 scRNA‐seq libraries across five groups: Intact (5 libraries), 3 days postinjury (dpi) control (CTRL; 2 libraries), 3 dpi inhibitor–treated (INH; 2 libraries), 5 dpi CTRL (3 libraries), and 5 dpi INH (3 libraries). No pain‐behavioral measurements were available in the original dataset. Therefore, the potential relevance of the identified transcriptional features to posttraumatic pain was evaluated using established pain‐relevant gene signatures, signaling pathways, and neuron–microglia communication patterns.

### 2.2. Data Processing and Batch Correction

The raw 10× Genomics gene‐expression matrices were processed using Seurat [24–27]. Potential doublets were identified and removed using DoubletFinder [28–30]. Cells with 300–6000 detected genes, 500–100,000 total unique molecular identifiers, mitochondrial gene expression below 25%, and hemoglobin‐gene expression below 5% were retained. The retained data were log‐normalized using NormalizeData with a scale factor of 10,000 and scaled using ScaleData. The top 2000 highly variable genes were identified using the variance‐stabilizing transformation method and used for principal component analysis [31, 32]. The first 30 principal components were retained, and Harmony was applied using sample identity as the batch variable [33].

### 2.3. Cell Clustering and Annotation

Harmony‐corrected embeddings were used for neighborhood construction, clustering, and uniform manifold approximation and projection (UMAP) visualization using the first 30 Harmony dimensions [34–36]. Major cell types were annotated using canonical marker genes, the CellMarker database, and published literature. Microglia were subsequently extracted and reclustered using the same analytical framework to identify transcriptionally distinct subpopulations. Observed‐to‐expected ratios (Ro/e) were calculated to assess the relative representation of cell populations across groups, with Ro/e > 1 indicating preferential representation.

### 2.4. Differential Gene Expression and Functional Enrichment Analysis

Cluster‐enriched marker genes were identified using Seurat’s FindAllMarkers function with the Wilcoxon rank‐sum test. Genes with an average log2 fold change > 0.25, expression in at least 25% of cells in the corresponding cluster, and a Benjamini–Hochberg‐adjusted *p* value < 0.05 were retained as positive marker genes [37]. Functional enrichment analyses were performed using clusterProfiler (v4.14.0). Gene Ontology (GO) enrichment analysis was used to characterize the biological processes and molecular functions associated with each cell population or microglial subpopulation. Gene set enrichment analysis (GSEA) was additionally performed using ranked gene‐expression results to identify coordinated transcriptional programs associated with individual microglial subpopulations [38–40].

### 2.5. AUCell Analysis

The scgmt package (v0.0.3; available at https://github.com/ZhaoLabs-SJTU/scgmt), which implements the AUCell algorithm, was used to calculate gene‐signature enrichment scores at the single‐cell level. AUCell scores reflect the relative enrichment of each gene signature within individual cells and were used for comparative analyses [41].

### 2.6. CytoTRACE and Slingshot Analysis

CytoTRACE (v0.3.3) was used to estimate relative transcriptional complexity among microglial subpopulations [42]. Slingshot (v2.10.0) was subsequently applied in a fully unsupervised manner to infer potential state‐transition trajectories [43]. Slingshot constructed a cluster‐level minimum spanning tree and identified the lineage structure and computational orientation using its default algorithm. Smooth lineage curves and lineage‐specific pseudotime values were generated using the getCurves function. The inferred pseudotime was interpreted as an ordering of transcriptional states rather than direct evidence of chronological progression or lineage differentiation [44].

### 2.7. Intercellular Communication Analysis

CellChat (v1.6.1) was used to infer potential intercellular communication across the integrated dataset [45]. Cells from all groups were pooled and classified according to their annotated major cell types and microglial subpopulations. Ligand–receptor interactions were evaluated using CellChatDB.mouse, including the secreted signaling, extracellular matrix‐receptor, and cell–cell contact categories. Communication probabilities were assessed using the permutation‐based framework implemented in CellChat, and interactions with *p* < 0.05 were retained. The resulting network represents the overall communication landscape across the pooled dataset rather than time‐ or treatment‐specific patterns [46–48].

### 2.8. Transcriptional Regulatory Network Analysis

Transcriptional regulatory network analysis was performed separately for the microglial datasets using pySCENIC [49]. GRNBoost2 was used to infer candidate transcription factor–target gene interactions, which were refined by motif enrichment analysis to define regulons. Regulon activity was quantified at the single‐cell level using AUCell. Regulon specificity score (RSS), calculated based on Jensen–Shannon divergence, were used to identify subpopulation‐associated regulons, and the five regulons with the highest RSS values in each microglial subpopulation were visualized. Mean AUCell scores were used to summarize overall regulon activity.

### 2.9. Statistical Analysis

**FIGURE 1:**
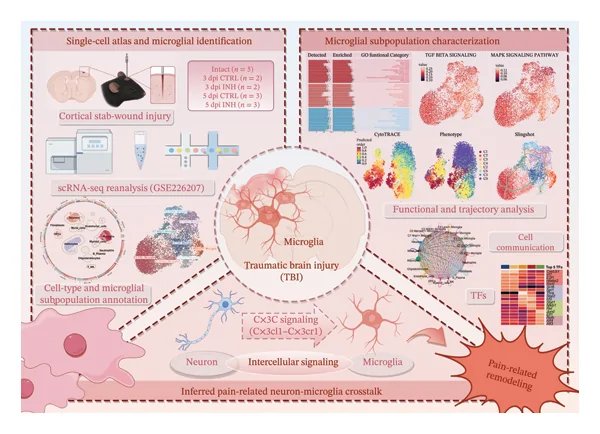
Overview of the research.

Statistical analyses were performed using R (v4.3.0) and Python (v3.9.0). Differential gene‐expression analyses were conducted using the Wilcoxon rank‐sum test implemented in Seurat. *p* values were adjusted for multiple comparisons using the Benjamini–Hochberg method, and an adjusted *P* value below 0.05 was considered statistically significant. CytoTRACE scores were compared among microglial subpopulations using the Wilcoxon rank‐sum test. Statistical significance of CellChat‐inferred ligand–receptor interactions was evaluated using permutation testing, with *p* < 0.05 considered significant. Analyses of cell abundance, Ro/e, and gene‐signature scores were interpreted in conjunction with their consistency across complementary analyses. The overall analytical workflow is summarized in Figure 1.

## 3. Results

### 3.1. Single‐Cell Transcriptomic Profiling Identifies Injury‐Associated Remodeling of the Cortical Cellular Landscape

After quality control, data integration, and batch‐effect correction, 66,310 cells from 15 intact and postinjury cortical scRNA‐seq libraries in GSE226207 were retained for downstream analysis. Unsupervised clustering and marker‐gene‐based annotation identified 12 major cell populations, including neurons, microglia, mural cells, astrocytes, oligodendrocytes, endothelial cells, oligodendrocyte precursor cells (OPCs), myeloid cells, T/NK cells, B/plasma cells, fibroblasts, and neutrophils (Figure 2A). Quality‐control metrics were summarized across the major cell populations and were consistent with the applied filtering criteria (Figure 2B).

FIGURE 2Single‐cell transcriptomic atlas reveals cellular heterogeneity after traumatic brain injury (TBI). (A) UMAP visualization of the major cell populations identified in the integrated cortical dataset. The concentric annotation tracks surrounding the embedding indicate the cluster identity, group, and cell‐cycle phase. The four surrounding feature plots show the distributions of nCount_RNA, nFeature_RNA, G2M.Score, and S.Score. (B) Violin plots comparing nCountRNA, nFeatureRNA, G2M.Score, and S.Score across the identified cell populations. (C) UMAP plots showing cellular distributions across 15 scRNA‐seq libraries, colored by sample identity (orig.ident), group, and cell‐cycle phase. (D) Dot plot showing the expression of the top five marker genes identified for each major cell population across cell populations (left) and groups (right). Dot size represents the percentage of cells expressing each gene, and color represents the z‐scored average expression. (E) Stacked bar plots showing the relative proportions of major cell populations across groups (top) and cell‐cycle phases (bottom). (F) Observed‐to‐expected (Ro/e) ratio heatmaps showing the relative enrichment of major cell populations across groups (left) and cell‐cycle phases (right). (G) Differential expression scatter plots comparing each major cell population with the remaining cells. Representative differentially expressed genes are labeled. (H) Gene Ontology (GO) functional classification of microglial differentially expressed genes, showing the numbers of detected and enriched genes assigned to representative molecular function (MF), cellular component (CC), and biological process (BP) categories. (I) Bar plot showing representative enriched GO‐BP terms associated with microglia. (J) Word cloud visualization summarizing enriched GO‐BP terms associated with microglia. (K) Gradient bar plot highlighting GO‐BP terms enriched in microglia. (L) Gene set enrichment analysis (GSEA) showing representative immune‐related GO‐BP terms enriched in microglia, including the immune effector process, regulation of the immune effector process, regulation of leukocyte‐mediated immunity, and regulation of the immune response.

**Figure:**
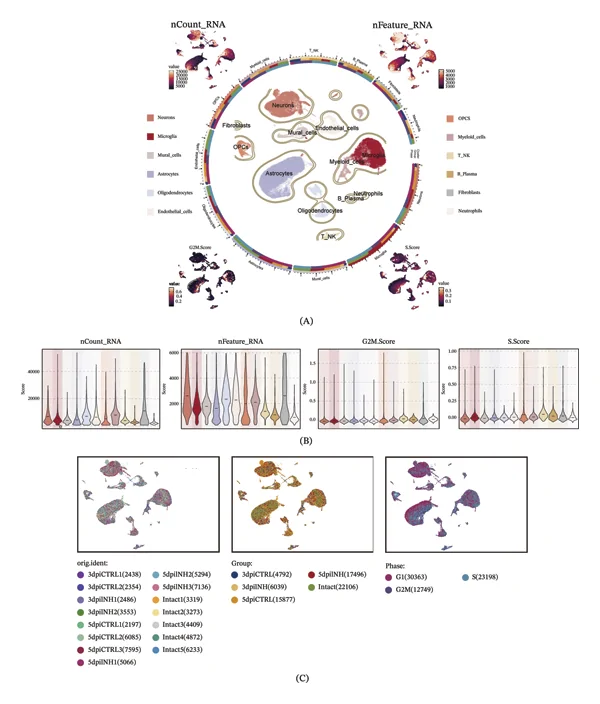

**Figure:**
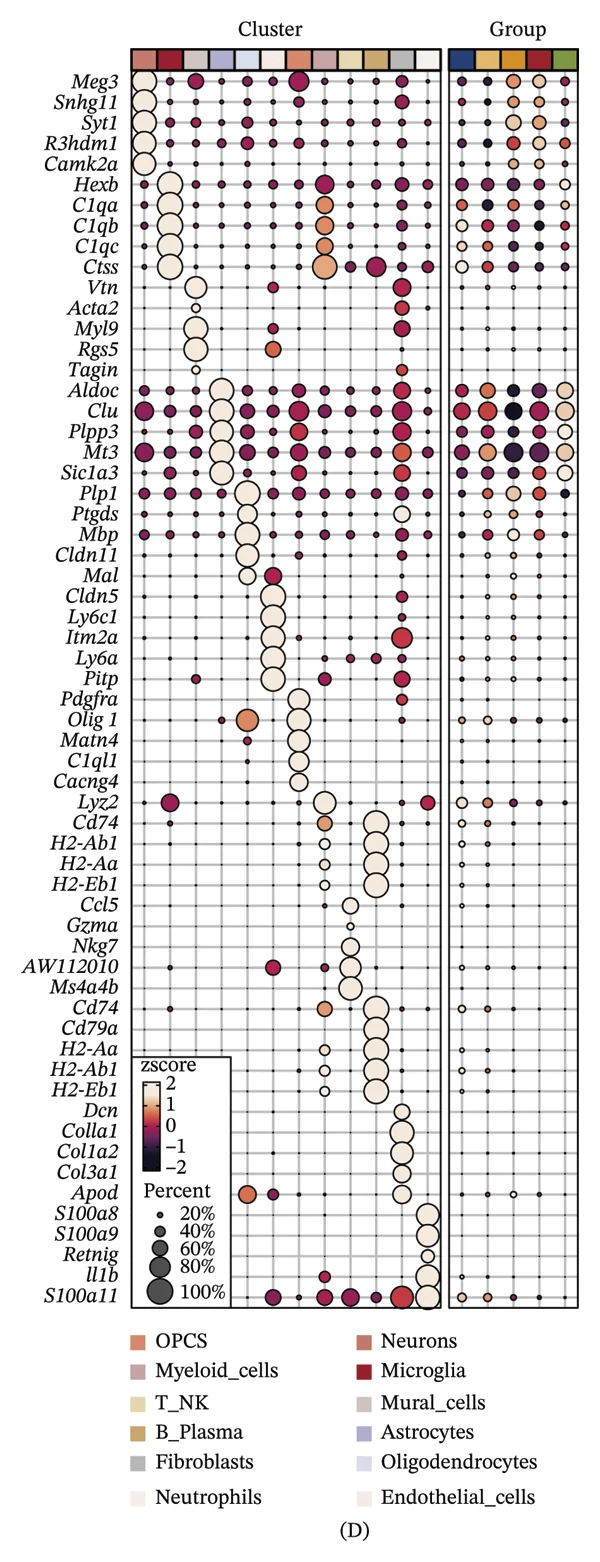

**Figure:**
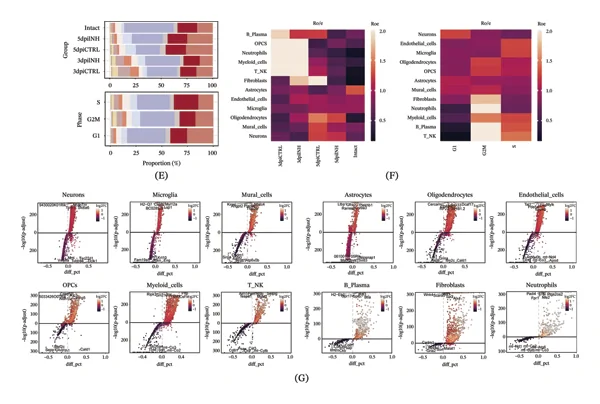

**Figure:**
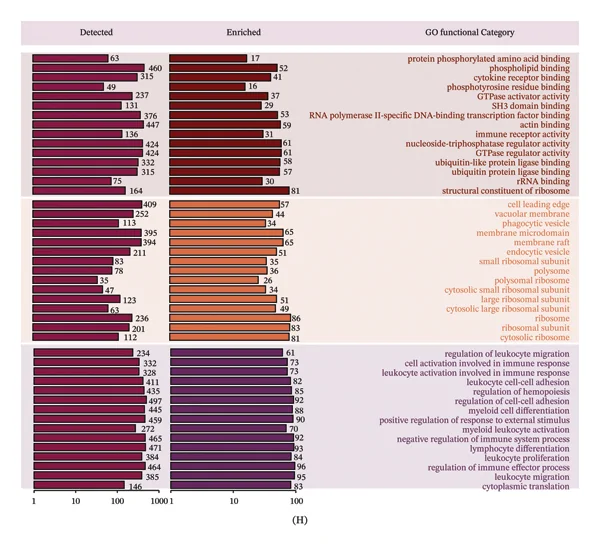

**Figure:**
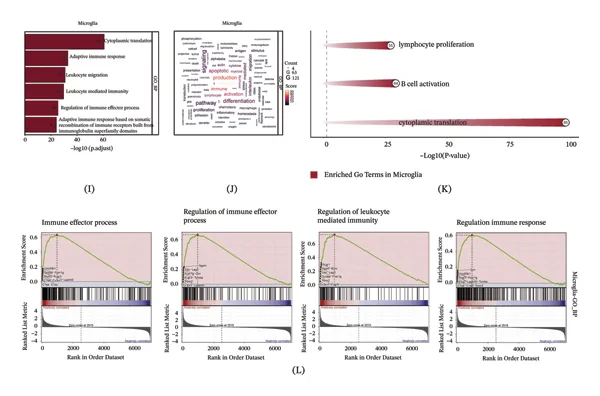

The integrated dataset comprised five groups defined by injury status, postinjury time point, and inhibitor treatment: Intact, 3 dpi CTRL, 3 dpi INH, 5 dpi CTRL, and 5 dpi INH. UMAP visualization showed the distribution of cells by sample, group, and cell‐cycle phase, with cells from different libraries broadly represented across the major clusters (Figure 2C). Dot plot analysis of representative marker genes further supported the cell‐type annotations and showed their expression patterns across groups (Figure 2D).

Astrocytes, neurons, and microglia were the predominant populations, although cell‐type composition varied among groups and cell‐cycle phases (Figure 2E). Ro/e analysis revealed condition‐dependent enrichment of OPCs and several immune cell populations, including neutrophils, myeloid cells, and T/NK cells, with the most pronounced differences observed at 3 dpi (Figure 2F). Cell cycle–based analysis showed relative enrichment of fibroblasts and several immune populations in the G2/M phase, whereas neurons were preferentially represented in G1, consistent with the limited proliferative activity of mature neurons.

Cell type–level differential expression analysis identified distinct transcriptional profiles across the major cortical populations (Figure 2G). Microglia showed relatively high expression of *H2-Q7, Calm2, Lsp1,* and *Myl12a*. Astrocytes were characterized by the enrichment of *Ltbp1, Gfra1,* and *Rarres2*, whereas oligodendrocytes showed relatively high expression of *Cercam* and *Zfp488*. Together with the markers shown in Figure 2D, these population‐associated profiles supported the annotation and transcriptional heterogeneity of the integrated dataset.

Functional enrichment analysis indicated that microglial genes were mainly enriched in immune‐related processes, including leukocyte activation, migration, and regulation of immune effector responses, together with cytoplasmic translation (Figures 2H–K). GSEA further showed the enrichment of immune effector processes and regulatory programs associated with immune effector activity, leukocyte‐mediated immunity, and broader immune responses in microglia (Figure 2L). These findings identified microglia as a prominent immune‐responsive compartment of the injured cortical environment and provided the basis for subsequent subpopulation‐level analyses in the context of post‐TBI pain.

### 3.2. Microglial Subclustering Identifies Eight Subpopulations and Reveals Condition‐Dependent Representation of *L* *a* *r* *s*2^+^ Microglia

A total of 7648 microglia were extracted from the integrated dataset and subjected to secondary clustering. Eight transcriptionally distinct microglial subpopulations were identified and named according to representative genes: C0 *K* *l* *f*2^+^, C1 *C* *s* *t*7^+^, C2 *T* *n* *f* ^+^, C3 *S* *o* *c* *s*3^+^, C4 *L* *a* *r* *s*2^+^, C5 *I* *f* *i*204^+^, C6 *M* *a* *r* *c* *k* *s* *l*1^+^, and C7 *N* *r* *i* *p*1^+^ microglia (Figure 3A). UMAP feature plots showed the expression patterns of the representative genes used to name these microglial subpopulations (Figure 3B). Quality‐control metrics were summarized across the eight subpopulations and were consistent with the applied filtering criteria (Figure 3C). Dot plot visualization of selected cluster‐associated genes further demonstrated both distinct and partially shared transcriptional patterns among the microglial subpopulations and groups (Figure 3D).

FIGURE 3Single‐cell transcriptomics reveals microglial heterogeneity after TBI. (A) UMAP visualization of eight transcriptionally distinct microglial subpopulations: C0 *K* *l* *f*2^+^, C1 *C* *s* *t*7^+^, C2 *T* *n* *f* ^+^, C3 *S* *o* *c* *s*3^+^, C4 *L* *a* *r* *s*2^+^, C5 *I* *f* *i*204^+^, C6 *M* *a* *r* *c* *k* *s* *l*1^+^, and C7 *N* *r* *i* *p*1^+^ microglia. (B) UMAP plots showing the expression distributions of the marker genes used to annotate the eight microglial subpopulations. (C) UMAP feature plots and violin plots showing the distributions of nCount_RNA, nFeature_RNA, G2M.Score, and S.Score across the integrated microglial dataset and individual microglial subpopulations. (D) Dot plot showing the expression of the top five marker genes identified for each microglial subpopulation across microglial subpopulations (left) and groups (right). Dot size represents the percentage of cells expressing each gene, and color represents the z‐scored average expression. (E) Word cloud visualizations summarizing enriched biological process terms for each microglial subpopulation. (F) GO functional classification of genes associated with C4 microglia, showing the numbers of detected and enriched genes assigned to representative molecular function (MF), cellular component (CC), and biological process (BP) categories. (G) Gene set enrichment analysis (GSEA) showing positive enrichment of cytoplasmic translation, peptide biosynthetic process, and amide biosynthetic process in C4 *Lars2* ^+^ microglia. (H) Ro/e ratio heatmaps showing the relative enrichment of microglial subpopulations across groups (top) and cell‐cycle phases (bottom). (I) Differential‐expression scatter plots comparing each microglial subpopulation with all remaining microglia. Representative differentially expressed genes are labeled.

**Figure:**
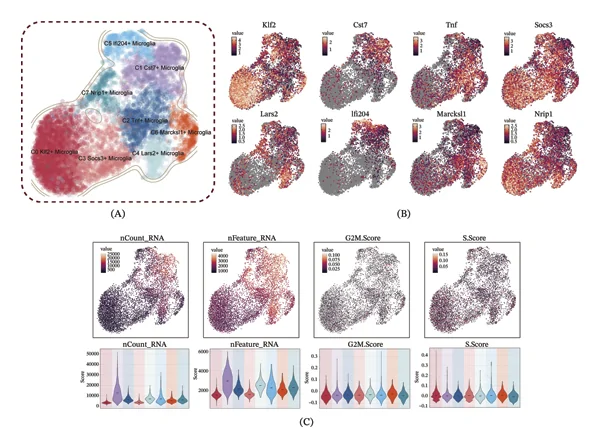

**Figure:**
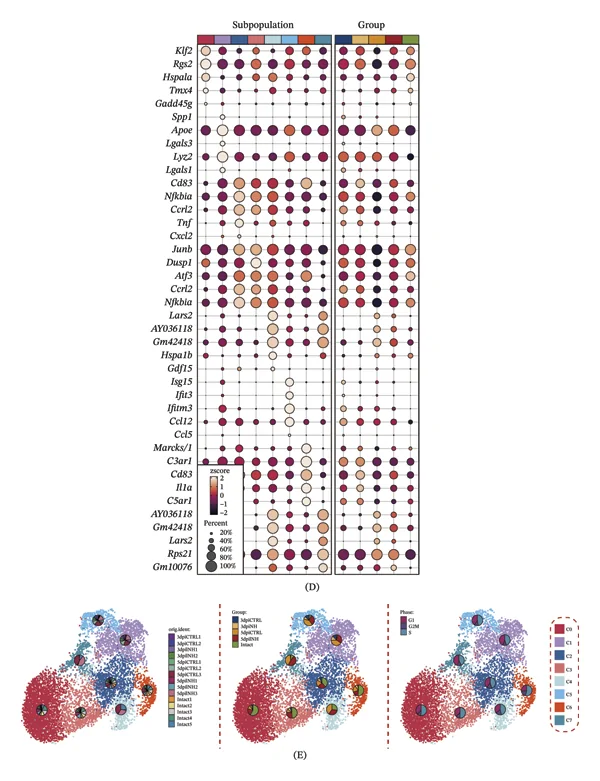

**Figure:**
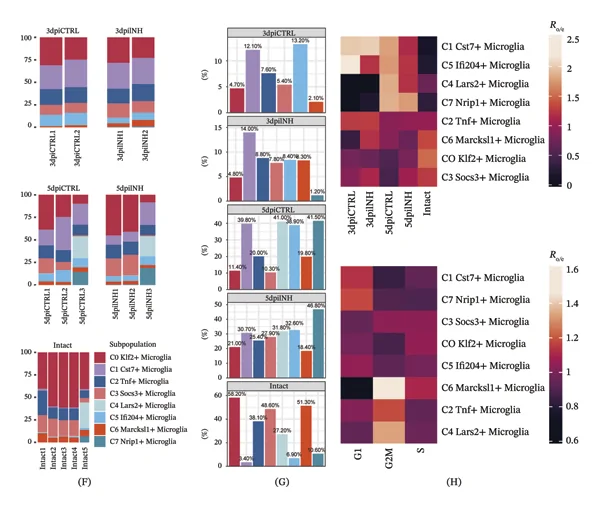

**Figure:**
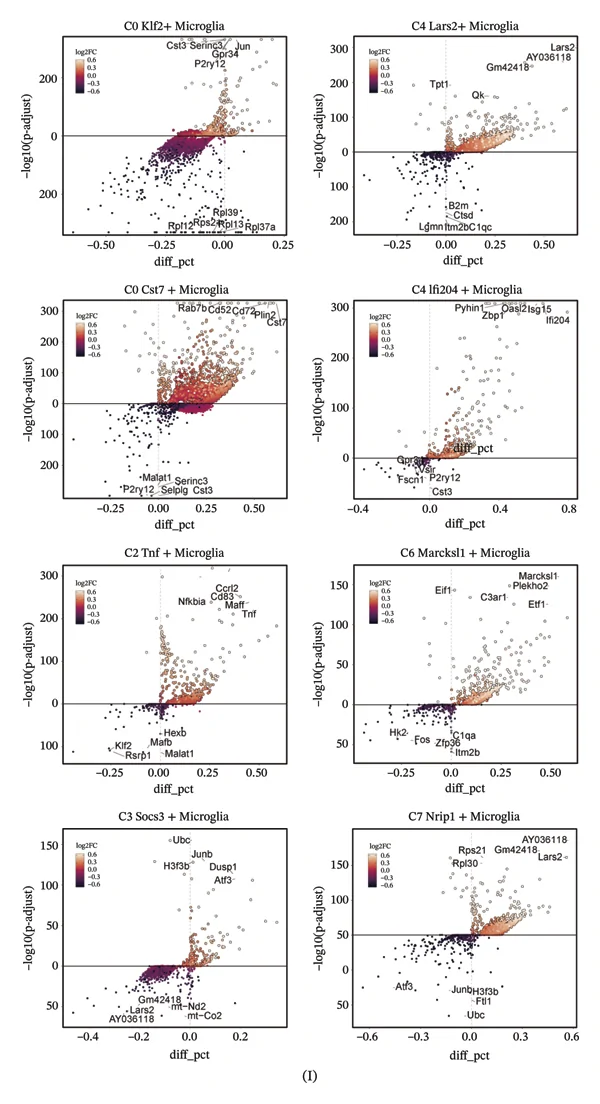

UMAP‐based pie‐chart overlays summarized the contributions of individual libraries, groups, and cell‐cycle phases to each microglial subpopulation (Figure 3E). Sample‐level stacked bar plots further showed a variation in subpopulation composition among biological replicates (Figure 3F). To examine the experimental group distribution of each subpopulation, the total number of cells assigned to each microglial subpopulation across the five groups was independently set to 100% (Figure 3G). Most C0 *K* *l* *f*2^+^ microglia were contributed by intact samples, whereas C1 *C* *s* *t*7^+^ and C5 *I* *f* *i*204^+^ microglia were derived predominantly from postinjury samples. C4 *L* *a* *r* *s*2^+^ microglia were contributed mainly by the 5 dpi CTRL, 5 dpi INH, and intact groups, accounting for 41.0%, 31.8%, and 27.2% of all C4 cells, respectively, with few cells contributed by the 3 dpi groups. A similar distribution was observed for C7 *N* *r* *i* *p*1^+^ microglia, most of which were derived from the 5 dpi groups. Consistent with these group‐composition profiles, Ro/e analysis further indicated underrepresentation of C4 *L* *a* *r* *s*2^+^ microglia in the 3 dpi groups and preferential representation in the 5 dpi groups, particularly in the 5 dpi CTRL condition (Figure 3H). Cell cycle–associated Ro/e also differed among the microglial subpopulations. C4 *L* *a* *r* *s*2^+^ and C6 *M* *a* *r* *c* *k* *s* *l*1^+^ microglia showed relatively greater representation in G2/M, whereas other subpopulations displayed different phase‐associated distributions.

Clusterwise differential expression analysis further delineated the transcriptional features of the eight microglial subpopulations (Figure 3I). C4 *L* *a* *r* *s*2^+^ microglia showed positive differential expression of *Lars2, AY036118, Gm42418, Tpt1,* and *Qk*, whereas *B2m*, *Ctsd*, *Lgmn*, *Itm2b*, and *C1qc* were relatively downregulated. Notably, *Lars2*, *AY036118*, and *Gm42418* were also positively associated with C7 *N* *r* *i* *p*1^+^ microglia, indicating partially shared transcriptional features between these subpopulations. Collectively, these analyses defined eight transcriptionally heterogeneous microglial states and identified condition‐ and time‐associated differences in the representation of C4 *L* *a* *r* *s*2^+^ microglia.

### 3.3. *L* *a* *r* *s*2^+^ Microglia Exhibit Neuroimmune and Neural Remodeling Programs Associated With Post‐TBI Pain

To characterize the functional heterogeneity of the identified microglial subpopulations, AUCell was used to evaluate proinflammatory and anti‐inflammatory gene signatures. Both signatures varied across the eight subpopulations, indicating heterogeneous inflammatory and immunoregulatory transcriptional states (Figure 4A). C4 *L* *a* *r* *s*2^+^ microglia showed intermediate scores for both signatures, without a marked bias toward either program. This profile suggested that C4 *L* *a* *r* *s*2^+^ microglia could not be readily classified according to a simple proinflammatory versus anti‐inflammatory framework.

FIGURE 4Functional characterization of microglial subpopulations and pain‐relevant signaling signatures. (A) UMAP plots and violin plots showing the distributions of proinflammatory and anti‐inflammatory gene‐signature scores across the eight microglial subpopulations. (B) Dot plot summarizing inflammatory, signaling, and pain‐related signature scores across microglial subpopulations and groups. (C) UMAP plots showing TGF‐β– and MAPK‐associated signature scores, with violin plots comparing their distributions across microglial subpopulations and groups. (D) UMAP plots and violin plots showing interferon‐γ response–, PI3K–AKT–mTOR‐, and IL‐6–JAK–STAT3‐associated signature scores across microglial subpopulations. (E) Word‐cloud visualizations summarizing enriched biological process terms for each microglial subpopulation. (F) GO functional classification of genes associated with C4 *L* *a* *r* *s*2^+^ microglia, showing the numbers of detected and enriched genes assigned to representative MF, CC, and BP categories. (G) GSEA showing positive enrichment of cytoplasmic translation, peptide biosynthetic process, and amide biosynthetic process in C4 *L* *a* *r* *s*2^+^ microglia.

**Figure:**
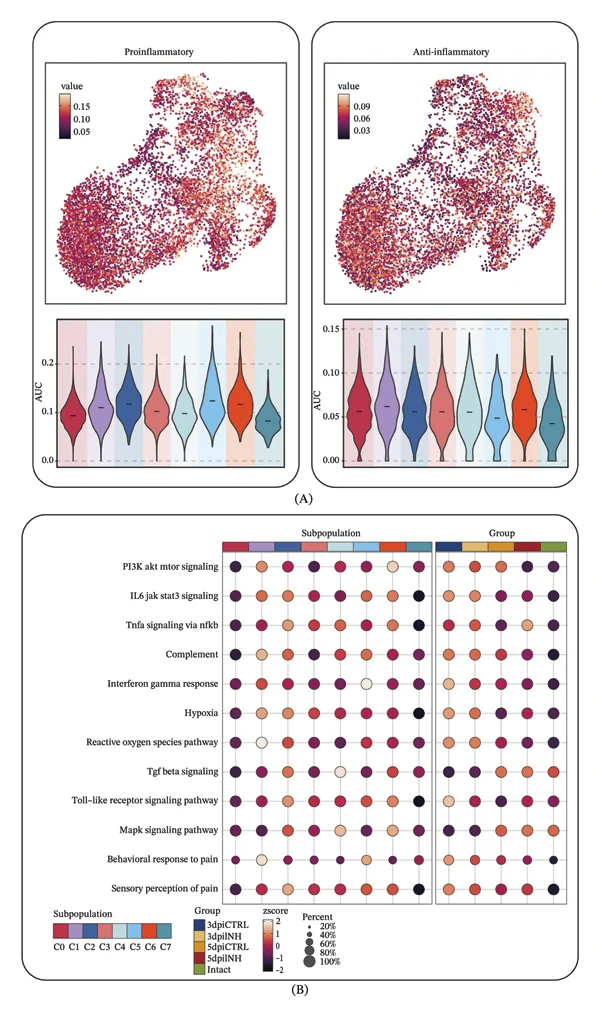

**Figure:**
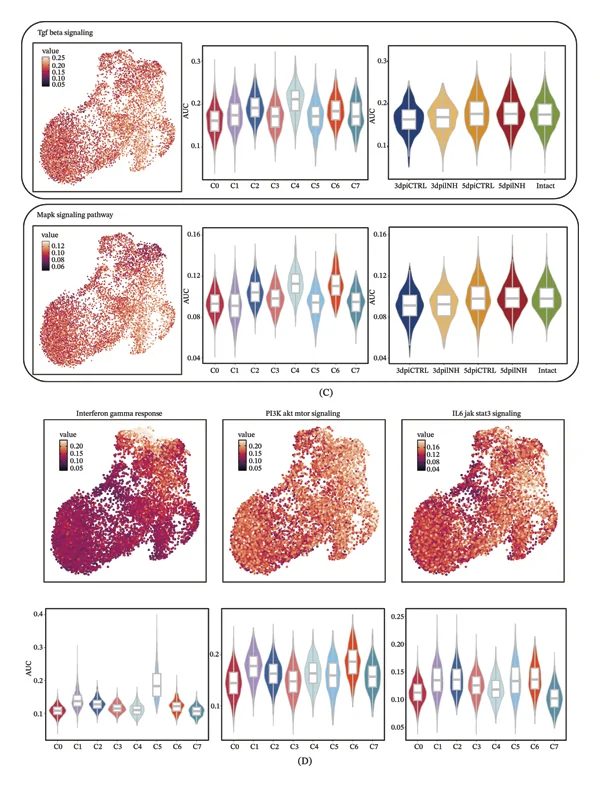

**Figure:**
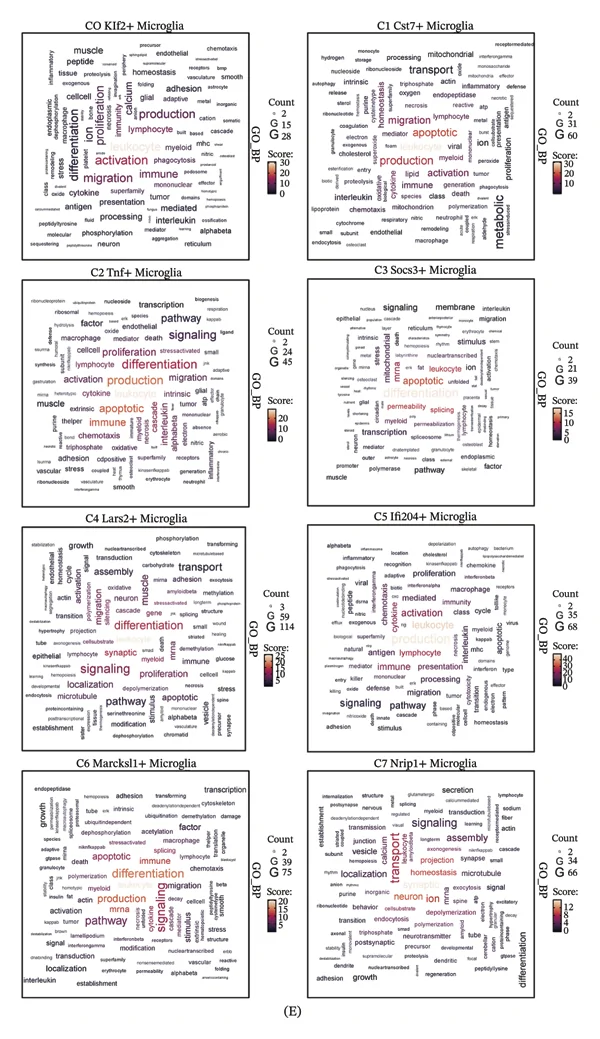

**Figure:**
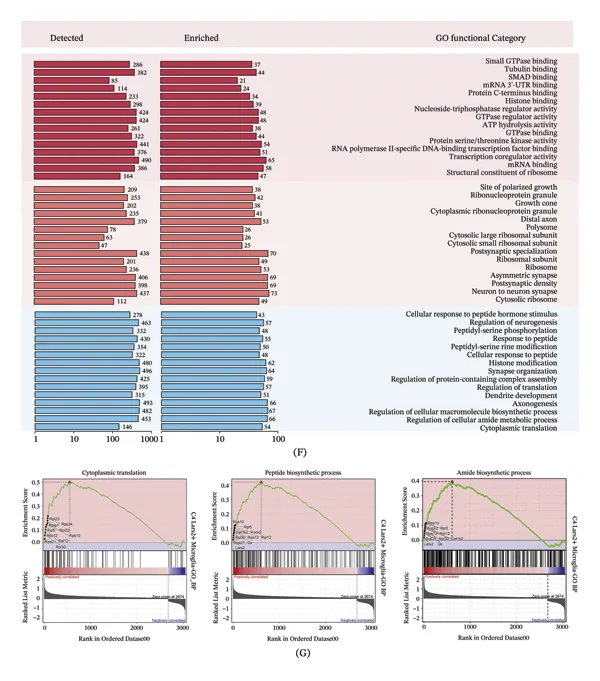

We next examined transcriptional programs potentially relevant to post‐TBI pain, including gene sets associated with sensory perception of pain, behavioral responses to pain, inflammatory signaling, cellular stress, and neural plasticity. Bubble plot analysis revealed distinct and partially overlapping patterns across the eight microglial subpopulations and groups (Figure 4B). In addition to pain‐associated gene sets, these patterns involved PI3K–AKT–mTOR, IL‐6–JAK–STAT3, TNF‐α–NF‐κB, complement, interferon‐γ response, hypoxia, reactive oxygen species, TGF‐β, Toll‐like receptor, and MAPK signaling. Overall, these programs showed heterogeneous distributions among microglial states, indicating that post‐TBI pain–associated responses involve multiple inflammatory, stress‐responsive, and signaling programs rather than a single pain‐specific state.

Although pain‐associated signatures were distributed across multiple microglial subpopulations, C4 *L* *a* *r* *s*2^+^ microglia exhibited a distinct transcriptional profile characterized by the enrichment of specific signaling programs. Specifically, C4 *L* *a* *r* *s*2^+^ microglia showed the highest TGF‐β–associated signature score and a relatively high MAPK‐associated signature score (Figure 4C). These features distinguished C4 *L* *a* *r* *s*2^+^ microglia from other subpopulations with more prominent inflammatory signatures, such as C5 *I* *f* *i*204^+^ and C6 *M* *a* *r* *c* *k* *s* *l*1^+^ microglia. At the group level, both signatures tended to be higher in the 5 dpi groups than in the 3 dpi groups, although comparable signals were also observed in the intact samples. These findings identify C4 *L* *a* *r* *s*2^+^ microglia as a distinct postinjury state characterized by TGF‐β– and MAPK‐associated transcriptional programs. Other subpopulations exhibited different signaling patterns, with C5 *I* *f* *i*204^+^ microglia showing the highest interferon‐γ response signature score, whereas C6 *M* *a* *r* *c* *k* *s* *l*1^+^ microglia showed relatively high PI3K–AKT–mTOR‐ and IL‐6–JAK–STAT3‐associated scores (Figure 4D).

Word‐cloud analysis summarized the functional heterogeneity of the eight microglial subpopulations (Figure 4E). Terms associated with C4 *L* *a* *r* *s*2^+^ microglia included synapse, leukocyte, differentiation, and proliferation. C5 *I* *f* *i*204^+^ microglia were associated with antigen presentation–related processes, whereas C6 *M* *a* *r* *c* *k* *s* *l*1^+^ microglia were linked to terms involving immunity, apoptosis, chemotaxis, NF‐κB signaling, and interferon‐associated processes. GO enrichment analysis of C4 *L* *a* *r* *s*2^+^ microglia further identified biological processes annotated to synaptic organization, axonogenesis, and dendrite development, together with molecular functions including SMAD binding (Figure 4F). GSEA additionally showed the enrichment of cytoplasmic translation, peptide biosynthetic process, and amide biosynthetic process gene sets (Figure 4G). These findings characterize C4 *L* *a* *r* *s*2^+^ microglia as a distinct postinjury state with neuroimmune and neural plasticity‐related transcriptional features that may be relevant to pain‐associated remodeling after TBI.

### 3.4. *L* *a* *r* *s*2^+^ Microglia Occupy a Distinct Position in the Inferred State Landscape

We next examined stemness‐associated transcriptional features and inferred state relationships among the eight microglial subpopulations. Although C3 *S* *o* *c* *s*3^+^ microglia showed the highest median score, C4 *L* *a* *r* *s*2^+^ microglia also displayed relatively high scores (Figures 5A and B). Several genes associated with cellular plasticity showed distinct expression patterns across subpopulations. Notably, C4 *L* *a* *r* *s*2^+^ microglia exhibited relatively higher expression of *Kdm5b*, *Notch1*, *Prom1*, *Ezh2*, and *Ctnnb1* compared with other microglial subpopulations (Figure 5C).

FIGURE 5Transcriptional heterogeneity and inferred trajectories of microglial subpopulations after TBI. (A) UMAP plot showing the distribution of stemness‐associated signature scores across individual microglial cells. (B) Violin plots comparing stemness‐associated signature scores among the eight microglial subpopulations. (C) Heatmap showing the scaled expression patterns of selected stemness‐associated genes across microglial subpopulations. (D) CytoTRACE analysis showing microglial subpopulation identity (top) and the predicted transcriptional ordering of individual microglial cells (bottom). (E) UMAP plot and box plots showing the distribution of CytoTRACE scores across microglial subpopulations. (F) Genes positively and negatively correlated with CytoTRACE scores. Bar length indicates the correlation coefficient. (G) Combined visualization of two Slingshot‐inferred lineages. (H) Separate visualization of Lineage 1 and Lineage 2. Arrows indicate the inferred pseudotime direction. (I) Sequential distributions of Lineage 1 and Lineage 2 along the inferred pseudotime. (J) Smoothed expression trends of representative microglial marker genes along inferred pseudotime. Points represent individual cells, and fitted curves show the lineage‐associated expression trends. (K) Smoothed expression trends of the stemness‐associated genes *Ctnnb1* and *Notch1* along inferred pseudotime.

**Figure:**
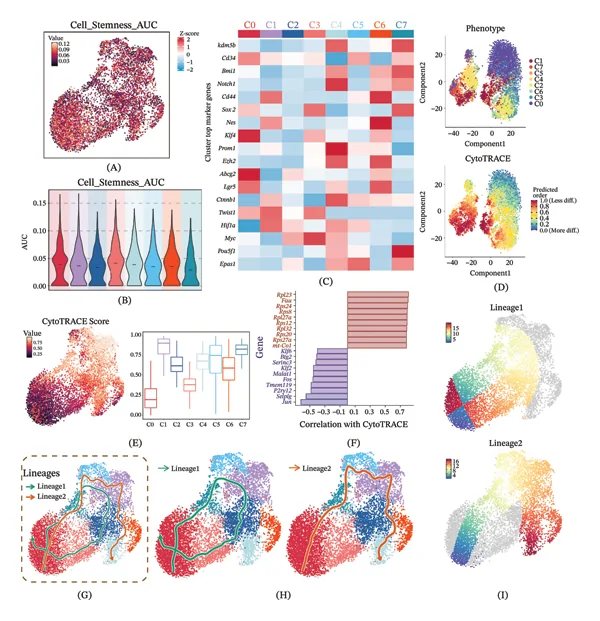

**Figure:**
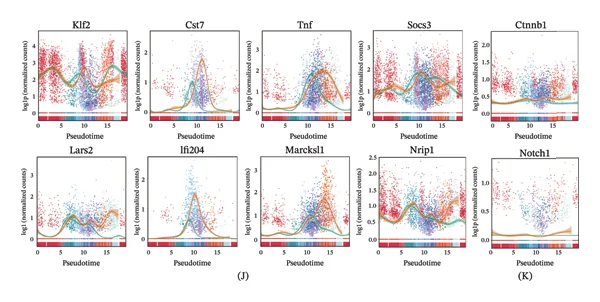

CytoTRACE analysis further revealed differences in the transcriptional state across the microglial subpopulations. C1 *C* *s* *t*7^+^ and C7 *N* *r* *i* *p*1^+^ microglia showed relatively high CytoTRACE scores, whereas C0 *K* *l* *f*2^+^ microglia showed the lowest score; C4 *L* *a* *r* *s*2^+^ microglia showed an intermediate‐to‐high score distribution (Figures 5D and E). This distribution suggests that C4 *L* *a* *r* *s*2^+^ microglia occupy a transcriptionally complex state distinct from both homeostatic and highly activated microglial populations. CytoTRACE scores were positively correlated with several ribosomal and mitochondrial transcripts, including *Rpl23*, *Rps24*, *Rps8*, and *mt-Co1*, and negatively correlated with the homeostatic microglial genes *Klf2*, *Tmem119*, and *P2ry12*, as well as the immediate‐early genes *Fos* and *Jun* (Figure 5F). Given the contribution of ribosomal and mitochondrial transcripts to these scores, CytoTRACE values were interpreted as exploratory indicators of transcriptional complexity rather than direct measures of developmental potential.

To further resolve relationships among microglial subpopulations, Slingshot analysis inferred two trajectories connecting the identified subpopulations (Figures 5G–I). Lineage 1 connected C0 *K* *l* *f*2^+^, C7 *N* *r* *i* *p*1^+^, C1 *C* *s* *t*7^+^, C2 *T* *n* *f* ^+^, and C3 *S* *o* *c* *s*3^+^ microglia. Lineage 2 connected C0 *K* *l* *f*2^+^, C7 *N* *r* *i* *p*1^+^, C5 *I* *f* *i*204^+^, C1 *C* *s* *t*7^+^, C2 *T* *n* *f* ^+^, C6 *M* *a* *r* *c* *k* *s* *l*1^+^, and C4 *L* *a* *r* *s*2^+^ microglia. Notably, C4 *L* *a* *r* *s*2^+^ microglia occupied the distal region of Lineage 2, representing a distinct transcriptional position within the inferred continuum and separating this population from upstream inflammatory states. Consistent with this position, fitted *Lars2* expression increased toward the distal portion of Lineage 2 (Figure 5J). *Ctnnb1* and *Notch1* also showed more modest increases toward the same region (Figure 5K).

### 3.5. CellChat Analysis Predicts a Neuron‐to‐Microglia CX3CL1–CX3CR1 Communication Axis Involving *L* *a* *r* *s*2^+^ Microglia

CellChat analysis was performed to infer communication patterns across the integrated dataset. Both the number and inferred strength of interactions indicated extensive communication among microglial subpopulations and other cell populations (Figure 6A). Heatmap analysis further revealed distinct outgoing and incoming signaling patterns across cell populations, indicating heterogeneous sender and receiver roles within the communication network (Figure 6B).

FIGURE 6Pooled CellChat analysis identifies potential intercellular communication involving *L* *a* *r* *s*2^+^ microglia. (A) Global communication networks showing the inferred number of interactions (left) and interaction strength (right) among microglial subpopulations and other cortical cell populations. (B) Heatmaps showing the relative outgoing (left) and incoming (right) signaling patterns across microglial subpopulations and other cell populations. Rows represent the signaling pathways, and columns represent the cell populations. (C) Circle plots showing the number of predicted interactions (left) and aggregate interaction strength (right) involving C4 *L* *a* *r* *s*2^+^ microglia. Upper panels represent outgoing signaling from C4 *L* *a* *r* *s*2^+^ microglia as the source population, whereas lower panels represent incoming signaling received by C4 *L* *a* *r* *s*2^+^ microglia as the target population. (D) Hierarchical network plot showing the inferred source–target relationships within the CX3C signaling pathway. (E) Dot plot showing predicted ligand–receptor interactions from different source cell populations to C4 *L* *a* *r* *s*2^+^ microglia. (F) Network‐centrality heatmap showing the predicted sender, receiver, mediator, and influencer roles of individual cell populations within the CX3C signaling network. (G) Dot plot showing the expression patterns of *Cx3cl1* and *Cx3cr1* across microglial subpopulations and other cell populations. Dot size represents the percentage of cells expressing each gene, and color represents scaled average expression. (H–I) Circular plot and chord diagram show the predicted communication network of the CX3CL1–CX3CR1 ligand–receptor pair.

**Figure:**
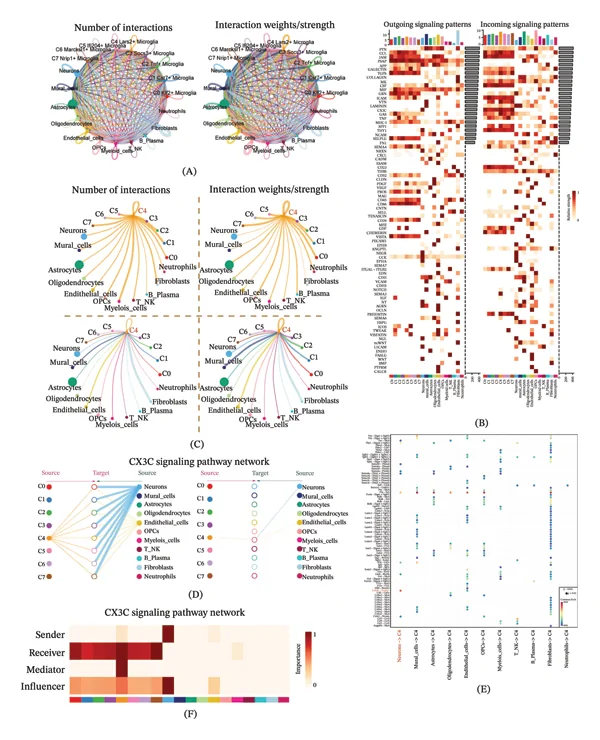

**Figure:**
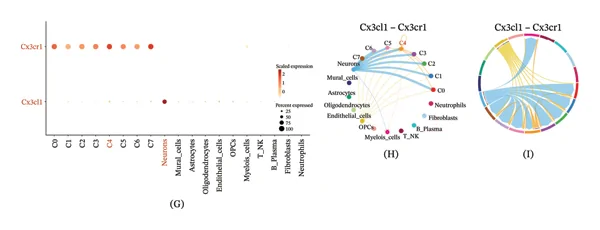

Given the enrichment of neural remodeling–related programs in C4 *L* *a* *r* *s*2^+^ microglia, we next examined their inferred communication landscape. C4 *L* *a* *r* *s*2^+^ microglia were embedded within a broad bidirectional communication network involving multiple microglial subpopulations, neurons, astrocytes, oligodendrocytes, OPCs, endothelial cells, mural cells, and immune cell populations. The number and inferred strength of interactions suggested that C4 *L* *a* *r* *s*2^+^ microglia potentially engaged in communication with diverse cellular compartments within the cortical microenvironment (Figure 6C).

We next asked whether neuron‐derived signals were associated with the C4 *L* *a* *r* *s*2^+^ microglial state through specific intercellular communication pathways. Analysis of the CX3C signaling pathway network revealed that neurons represented a major predicted source of CX3C signals, with higher inferred interactions directed toward microglial populations, including C4 *L* *a* *r* *s*2^+^ microglia (Figure 6D). To specifically examine signals received by C4 *L* *a* *r* *s*2^+^ microglia, we further analyzed C4 *L* *a* *r* *s*2^+^ microglia as the target population. Dot plot visualization showed multiple ligand–receptor interactions between neurons and C4 *L* *a* *r* *s*2^+^ microglia, among which neuron‐derived CX3C signaling represented a prominent predicted communication pattern (Figure 6E). Network‐centrality analysis assigned neurons a prominent sender role, while the microglial subpopulations showed relatively high receiver importance within the inferred CX3C network (Figure 6F).

Consistent with this network architecture, *Cx3cl1* expression was mainly detected in neurons, whereas *Cx3cr1* was broadly expressed across the microglial subpopulations, including C4 *L* *a* *r* *s*2^+^ microglia (Figure 6G). Among the predicted ligand–receptor interactions involving C4 *L* *a* *r* *s*2^+^ microglia, neuron‐derived CX3CL1 and microglial CX3CR1 showed a relatively high inferred communication probability (Figures 6H and I). Thus, these analyses predict a neuron‐to‐microglia CX3CL1–CX3CR1 communication axis involving C4 *L* *a* *r* *s*2^+^ microglia, suggesting that neuron‐derived signals may contribute to the cellular context associated with this remodeling‐related microglial state.

### 3.6. C4 *L* *a* *r* *s*2^+^ Microglia Exhibit a *Thra*‐Associated Regulatory Program

SCENIC analysis revealed distinct regulon activity profiles across the eight microglial subpopulations. Clustering of the connectivity specificity index matrix grouped the identified regulons into four modules, M1–M4 (Figure 7A). Projection of module activity onto the UMAP showed overlapping but distinct distributions across the microglial landscape (Figure 7B). Within M1, *Stat1* and *Stat2* showed the greatest variation across subpopulations, followed by regulons including *Elk3*, *Irf2*, *Elf1*, *Pura*, and *Thra* (Figure 7C).

FIGURE 7Regulon analysis reveals condition‐ and subpopulation‐associated regulatory programs in microglia. (A) Connectivity specificity index matrix showing the similarity of regulon‐activity patterns and the clustering of regulons into four regulatory modules (M1–M4). These module labels are unrelated to the conventional M1‐/M2‐like microglial polarization terminology. (B) UMAP plots showing the distribution of mean regulon activity for modules M1–M4 across individual microglial cells. (C) Rank plots showing the fraction of regulon‐activity variance across microglial subpopulations for modules M1–M4. (D) Comparison of M1–M4 module activity across groups. Scatter plots show group‐level regulon activity scores, and box plots show the distributions of module AUCell scores across the Intact, 3 dpi CTRL, 3 dpi INH, 5 dpi CTRL, and 5 dpi INH groups. (E) Comparison of M1–M4 module activity across the eight microglial subpopulations. Scatter plots show subpopulation‐level regulon activity scores, and box plots show the distributions of module AUCell scores across subpopulations. (F) Heatmap showing the nonredundant union of the top five regulons identified separately for each experimental group. Box plots compare the regulon activity of selected regulons across the Intact, 3 dpi CTRL, 3 dpi INH, 5 dpi CTRL, and 5 dpi INH groups. (G) Heatmap showing the nonredundant union of the top five regulons identified separately for each microglial subpopulation. (H) Regulon specificity score rankings for each microglial subpopulation. The five highest‐ranked regulons in each subpopulation are highlighted and labeled. (I) UMAP plots showing the single‐cell activity distributions of the top five regulons associated with the C4 *L* *a* *r* *s*2^+^ microglial subpopulation: *Pura, Thra, Egr2, Nr3c1,* and *Irf4*. (J) Box plots comparing the AUCell activity scores of these five regulons across the eight microglial subpopulations.

**Figure:**
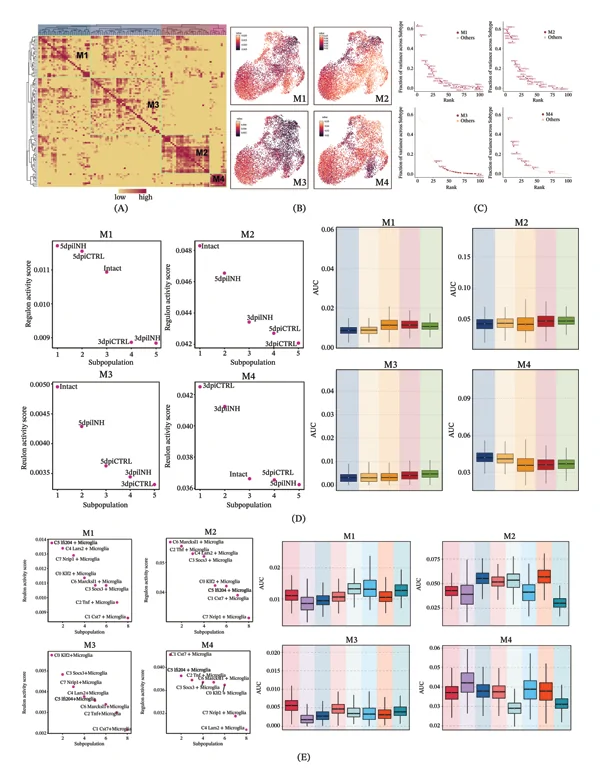

**Figure:**
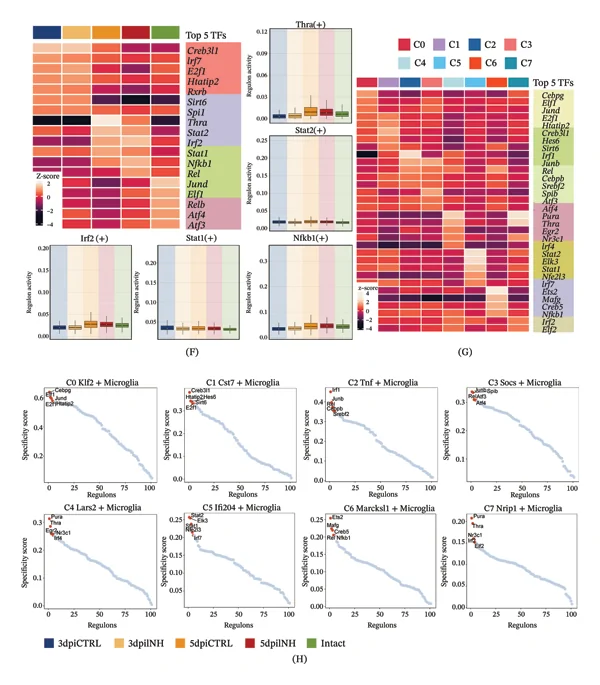

**Figure:**
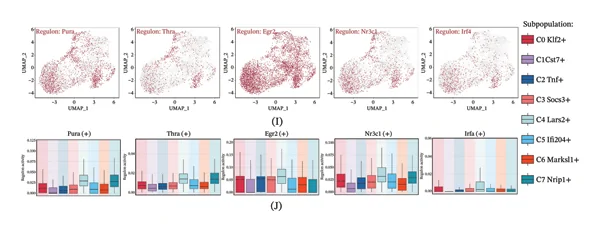

Module activity differed across experimental conditions. M1 activity was markedly increased in the 5 dpi groups, with the highest activity observed in the 5 dpi INH group, followed by the 5 dpi CTRL group, whereas both 3 dpi groups exhibited a lower M1 activity. Intact samples maintained an intermediate M1 activity level (Figure 7D). The remaining modules showed different group‐associated distributions, further supporting a condition‐dependent variation in microglial regulatory programs. Across microglial subpopulations, M1 activity was highest in C5 *I* *f* *i*204^+^ microglia and was also relatively high in C4 *L* *a* *r* *s*2^+^ and C7 *N* *r* *i* *p*1^+^ microglia (Figure 7E). By contrast, the activity patterns of Modules M2–M4 differed across the microglial subpopulations, indicating that individual subpopulations were associated with distinct combinations of regulatory modules rather than a single uniformly dominant program.

We next compared group‐associated regulon activity patterns. The five highest‐ranked regulons were identified separately for each experimental group, and duplicate regulons were removed to generate a nonredundant set for cross‐group comparison (Figure 7F). Within this set, *Thra*, *Stat2*, *Irf2*, *Stat1*, and *Nfkb1* showed a relatively prominent activity in the 5 dpi groups, although their rankings and activity distributions differed between the 5 dpi CTRL and 5 dpi INH conditions. These findings suggest that the 5 dpi response involved coordinated immune‐ and transcription‐associated regulatory programs.

We subsequently compared subpopulation‐associated regulon‐activity patterns. The five highest‐ranked regulons were identified separately for each microglial subpopulation and collectively visualized in a heatmap (Figure 7G). RSS analysis further ranked *Pura, Thra, Egr2, Nr3c1,* and *Irf4* as the top five regulons associated with the C4 *L* *a* *r* *s*2^+^ microglia (Figure 7H). The single‐cell distributions of these five regulons were subsequently visualized in UMAP space, and their activity patterns across all microglial subpopulations were compared using box plots (Figures 7I and J). Notably, the *Thra* regulon exhibited a relatively high activity in both *L* *a* *r* *s*2^+^ microglia and the 5 dpi groups. This shared pattern suggests that *Thra*‐associated transcriptional regulation may contribute to the regulatory profile of the C4 *L* *a* *r* *s*2^+^ microglia during the 5 dpi response.

## 4. Discussion

Pain after TBI remains a major clinical challenge and has been linked to persistent neuroinflammation, glial activation, altered neuronal excitability, and synaptic plasticity [13, 50, 51]. Microglia are among the first resident cells of the central nervous system to respond to brain injury and undergo marked transcriptional and functional changes that shape the evolving secondary injury response [52, 53]. An unresolved question is therefore not simply whether microglia are activated after TBI, but which microglial subpopulations predominate at different stages and how their transcriptional programs relate to pain‐relevant neural plasticity. Although astrocytes and oligodendrocyte‐lineage cells also contribute to post‐TBI inflammation, metabolic support, myelin homeostasis, and circuit remodeling [54, 55], microglia were prioritized here because of their rapid injury response and direct involvement in neuroimmune signaling and synaptic regulation [52, 56]. Single‐cell transcriptomic analysis resolved eight transcriptionally distinct microglial subpopulations after cortical stab‐wound injury. Among them, C4 *L* *a* *r* *s*2^+^ microglia were distinguished by TGF‐β– and MAPK‐associated signatures, enrichment of neural remodeling programs, a distinct position within the inferred transcriptional landscape, predicted neuronal CX3CL1–CX3CR1 input, and *Thra*‐associated regulon activity. This profile places C4 *L* *a* *r* *s*2^+^ microglia outside a simple pro‐ or anti‐inflammatory classification and identifies them as a candidate subpopulation relevant to posttraumatic neuroimmune and neural remodeling processes.

C4 *L* *a* *r* *s*2^+^ microglia were sparsely represented in the 3 dpi groups but showed greater representation at 5 dpi, with Ro/e analysis indicating preferential enrichment in the 5 dpi CTRL condition. This temporal distribution supports stage‐dependent heterogeneity in the microglial response to traumatic cortical injury [16]. The 5 dpi interval coincides with ongoing glial reorganization and evolving neuronal adaptation, suggesting that the increased representation of C4 *L* *a* *r* *s*2^+^ microglia may be associated with early processes relevant to pain‐related plasticity. Functional signatures also varied across microglial subpopulations. *I* *f* *i*204^+^ microglia were characterized predominantly by interferon‐ and inflammation‐associated features, whereas *M* *a* *r* *c* *k* *s* *l*1^+^ microglia showed relatively high PI3K–AKT–mTOR‐ and IL‐6–JAK–STAT3‐associated scores. These differences indicate that injury‐related neuroimmune and pain‐relevant programs are distributed across multiple microglial subpopulations rather than organized as a uniform response. Within this heterogeneous landscape, C4 *L* *a* *r* *s*2^+^ microglia did not exhibit a simple pro‐ or anti‐inflammatory bias but instead showed relatively high TGF‐β– and MAPK‐associated signature scores. TGF‐β–related programs may support microglial homeostasis, immune regulation, and tissue repair and may also participate in synaptic plasticity and remodeling [57–60], whereas MAPK signaling links injury‐responsive inflammation with neural plasticity, including synaptic reorganization, altered neuronal excitability, and central sensitization [61, 62]. Their coenrichment in C4 *L* *a* *r* *s*2^+^ microglia is therefore consistent with a potential role at the interface between postinjury neuroimmune regulation and pain‐related neural remodeling.

Functional enrichment analysis provided further support for the association of C4 *L* *a* *r* *s*2^+^ microglia with neural remodeling. This subpopulation was enriched for biological processes related to synaptic organization, axonogenesis, and dendrite development, together with cytoplasmic translation, peptide biosynthesis, and amide biosynthesis. Persistent pain is increasingly understood to involve maladaptive neural plasticity rather than inflammation alone [63, 64]. Microglia can influence synaptic maintenance, pruning, neuronal connectivity, and activity‐dependent plasticity, thereby contributing to the formation and persistence of sensitized neural circuits [65, 66]. The enrichment of synaptic and neurite‐related programs in C4 *L* *a* *r* *s*2^+^ microglia is therefore compatible with their involvement in cellular processes accompanying cortical circuit reorganization. The concurrent enrichment of translational and biosynthetic programs may further reflect increased protein‐synthesis demands during cellular adaptation. These transcriptional associations provide a basis for experimentally examining the role of C4 *L* *a* *r* *s*2^+^ microglia in pain‐relevant cortical remodeling.

CytoTRACE and Slingshot analyses placed C4 *L* *a* *r* *s*2^+^ microglia in a distinct region of the inferred transcriptional landscape. This subpopulation showed relatively high CytoTRACE scores and occupied the distal region of one Slingshot‐inferred lineage. The distal position of C4 *L* *a* *r* *s*2^+^ microglia, together with the fitted expression patterns of *Lars2*, *Notch1*, and *Ctnnb1*, indicates that this subpopulation has diverged transcriptionally from several other microglial populations. Notch and Wnt/β‐catenin signaling have been implicated in cellular plasticity, neural remodeling, and neuropathic pain [67, 68]. Although the present data do not establish the activation of these pathways or lineage progression, they support a distinct transcriptional configuration of C4 *L* *a* *r* *s*2^+^ microglia that is compatible with pain‐related neural remodeling.

The transcriptional distinction of C4 *L* *a* *r* *s*2^+^ microglia may reflect not only intrinsic regulatory programs but also signals received from the surrounding cortical environment. CellChat analysis placed this subpopulation within a broad communication network involving neurons, astrocytes, OPCs, and myeloid populations. Because cells from all groups were pooled, this network represents the overall communication architecture of the integrated dataset and cannot resolve interactions specific to injury stage or inhibitor treatment. Within the inferred CX3C network, neurons were positioned predominantly as signal senders, whereas several microglial subpopulations, including C4 *L* *a* *r* *s*2^+^ microglia, were positioned mainly as recipients.

The CX3CL1–CX3CR1 axis is a well‐established route of neuron–microglia communication and contributes to microglial surveillance, synaptic regulation, and context‐dependent inflammatory responses [69, 70]. In the present dataset, *Cx3cl1* expression was concentrated in neurons, whereas *Cx3cr1* was broadly expressed across microglial subpopulations. Together with the CellChat‐predicted interaction involving C4 *L* *a* *r* *s*2^+^ microglia, this expression pattern identifies a plausible route through which neuronal signals may influence this subpopulation. CX3CL1–CX3CR1 signaling is not uniformly proinflammatory or pain‐promoting; its effects vary with anatomical location, injury stage, and the surrounding inflammatory environment [70, 71]. Nevertheless, altered signaling through this axis has been linked to synaptic remodeling, neuronal excitability, and pain sensitization [72, 73]. When considered alongside the neural remodeling programs enriched in C4 *L* *a* *r* *s*2^+^ microglia, the predicted CX3CL1–CX3CR1 interaction provides a mechanistically coherent connection between cortical injury, neuronal input, and pain‐related neural plasticity.

SCENIC analysis further strengthened the distinction of C4 *L* *a* *r* *s*2^+^ microglia by identifying a characteristic regulatory program associated with this subpopulation. Although M1 activity was shared across several microglial subpopulations, regulon specificity analysis linked *Pura*, *Thra*, *Egr2*, *Nr3c1*, and *Irf4* most strongly to C4 *L* *a* *r* *s*2^+^ microglia. Among these regulons, *Thra* was particularly notable because its activity was elevated both in C4 *L* *a* *r* *s*2^+^ microglia and in the 5 dpi groups, coinciding with the time point at which this subpopulation became more abundant. This temporal‐ and subpopulation‐level concordance places *Thra*‐associated regulation within the broader molecular program that distinguishes C4 *L* *a* *r* *s*2^+^ microglia during the 5 dpi response. Thra encodes thyroid hormone receptor *α*1 (TRα1), a nuclear receptor that mediates thyroid hormone–dependent transcription and contributes to neural development [74, 75]; more broadly, thyroid hormone signaling has been implicated in microglial maturation, migration, and phagocytic activity [75–77]. Thus, the *Thra*‐associated regulon may reflect a transcriptional program relevant to the functional adaptation of C4 *L* *a* *r* *s*2^+^ microglia during the 5 dpi response. Together with the mitochondria‐associated transcripts and biosynthetic programs enriched in this subpopulation, these findings suggest coordinated regulatory and metabolic features.

The anatomical context of the present study should also be considered. The analyzed samples were obtained from injured somatosensory cortical gray matter. The primary somatosensory cortex is not simply a passive recipient of nociceptive input; recent work has shown that distinct cortical output pathways can bidirectionally regulate sensory gain and nociceptive behavior [78]. Following TBI, microglia can sustain cortical inflammation and neuronal dysfunction, while increased microglia–neuron contact and synaptic engulfment have been associated with synaptic loss and altered inhibitory transmission [15, 79]. More directly, selective activation of cortical microglia in the primary somatosensory cortex was recently shown to increase neuronal activity and induce pain hypersensitivity in mice [80]. These findings support the possibility that injury‐associated cortical inflammation and microglia‐related synaptic remodeling contribute to posttraumatic pain through altered cortical excitability and network plasticity. The identification of C4 *L* *a* *r* *s*2^+^ microglia in injured cortical gray matter therefore provides a plausible cellular link between local neuroimmune remodeling and the cortical processing of post‐TBI pain. Microglial activation and pain‐related functions have also been reported in the thalamus, periaqueductal gray, and spinal dorsal horn [81–83]. Whether transcriptionally similar C4 *L* *a* *r* *s*2^+^ microglia occur in these pain‐processing regions remains to be determined.

Overall, our data identify C4 *L* *a* *r* *s*2^+^ microglia as a temporally enriched subpopulation that may link early posttraumatic neuroimmune responses to pain‐relevant cortical remodeling. Their greater representation at 5 dpi coincided with TGF‐β– and MAPK‐associated signatures, enrichment of synaptic and axonal programs, predicted neuronal CX3CL1–CX3CR1 input, and *Thra*‐associated regulon activity. The convergence of these independent features is not readily accommodated by the conventional M1/M2 framework and refines the broad concept of posttraumatic microglial activation to a defined subpopulation, postinjury interval, and set of candidate molecular interactions. *L* *a* *r* *s*2^+^ microglia should therefore not be viewed as a pain‐specific population, but as a tractable cellular context in which neuroinflammation, neuronal communication, and circuit plasticity may intersect. These findings also argue for a more selective approach to studying microglial contributions to post‐TBI pain. Broad suppression of microglial activation may obscure functions that are restricted to particular subpopulations or stages of injury.

These translational implications should, however, be interpreted in light of several limitations. The original dataset did not include pain‐behavioral assessments, so the relationship between C4 *L* *a* *r* *s*2^+^ microglia and pain was inferred from transcriptional signatures, neural remodeling programs, and pain‐relevant signaling pathways rather than demonstrated directly. The available samples covered only 3 and 5 dpi, which limited the evaluation of later changes associated with persistent pain. In addition, the cortical stab‐wound model and gray‐matter sampling may not capture the full range of responses across other TBI models or pain‐processing regions. The small and unequal sample sizes, particularly at 3 dpi, also restricted group‐level comparisons. Because CellChat analysis was performed on cells pooled from all groups, the inferred network reflects the overall communication landscape and does not resolve time‐ or treatment‐specific changes. All animals were male, precluding the assessment of sex‐related differences. Further validation using independent datasets, spatial and protein‐level analyses, targeted functional experiments, and pain‐behavior testing will be needed to clarify the contribution of C4 *L* *a* *r* *s*2^+^ microglia to post‐TBI pain.

## 5. Conclusion

In conclusion, this study identifies the *L* *a* *r* *s*2^+^ microglial subpopulation as a temporally dynamic and transcriptionally specialized component of the postinjury microglial landscape. The convergence of pain‐relevant signaling signatures, neural remodeling programs, inferred state‐transition characteristics, CX3CL1–CX3CR1‐associated neuron–microglia communication, and *Thra*‐associated regulon activity supports a potential role for *L* *a* *r* *s*2^+^ microglia in posttraumatic neuroimmune and synaptic remodeling. These findings provide a cellular and molecular framework for future investigation of microglia‐targeted strategies for post‐TBI pain.

## Author Contributions

Fu Zhao led the conception and design of the study, coordinated the overall work, and took primary responsibility for manuscript preparation and revision. Fu Zhao, Jingru Shi, and Luxi Cao contributed to the study design. Fu Zhao and Jingru Shi drafted the initial manuscript. Fu Zhao and Yimin Zhang performed the statistical analysis and data processing. Yimin Zhang and Luxi Cao supervised the study and critically revised the manuscript. Fu Zhao and Jingru Shi contributed equally to this work.

## Funding

This study was supported by the National Natural Science Foundation of China (No.82174483) and the Natural Science Foundation of Guangdong Province (No. 2024A1515012169).

## Disclosure

All authors reviewed and approved the final version of the manuscript.

## Ethics Statement

This study involved only the reanalysis of publicly available scRNA‐seq data. No new animal experiments were conducted in the present study, and all animal procedures from the original dataset were performed with appropriate ethical approval.

## Conflicts of Interest

The authors declare no conflicts of interest.

## Data Availability

The data that support the findings of this study are openly available in Gene Expression Omnibus at https://www.ncbi.nlm.nih.gov/geo/, Ref. GSE226207.

## References

[1] Maas A. I. R. , Menon D. K. , Manley G. T. et al., Traumatic Brain Injury: Progress and Challenges in Prevention, Clinical Care, and Research, The Lancet. Neurology. (2022) 21, no. 11, 1004–1060, 10.1016/s1474-4422(22)00309-x. 36183712 PMC10427240

[2] Gbd T. B. I. A. , Global, Regional, and National Burden of Traumatic Brain Injury and Spinal Cord Injury, 1990-2016: A Systematic Analysis for the Global Burden of Disease Study 2016, The Lancet. Neurology. (2019) 18, no. 1, 56–87. 30497965 10.1016/S1474-4422(18)30415-0 PMC6291456

[3] Menon D. K. , Schwab K. , Wright D. W. , and Maas A. I. , Position Statement: Definition of Traumatic Brain Injury, Archives of Physical Medicine and Rehabilitation. (2010) 91, no. 11, 1637–1640, 10.1016/j.apmr.2010.05.017. 21044706

[4] Chen Q. , Bharadwaj V. , Irvine K. A. , and Clark J. D. , Mechanisms and Treatments of Chronic Pain After Traumatic Brain Injury, Neurochemistry International. (2023) 171, 10.1016/j.neuint.2023.105630. PMC11790307 37865340

[5] Nanda U. , Zhang G. , Underhill D. , and Pangarkar S. , Management of Pain and Headache After Traumatic Brain Injury, Physical Medicine and Rehabilitation Clinics of North America. (2024) 35, no. 3, 573–591, 10.1016/j.pmr.2024.02.009. 38945652

[6] Curran M. C. , Lucas S. , Fann J. R. , Zumsteg J. M. , and Hoffman J. M. , Chronic Pain After Traumatic Brain Injury: A Collaborative Care Approach, Frontiers in rehabilitation sciences. (2024) 5, 10.3389/fresc.2024.1398856. PMC11381419 39253025

[7] Sergeyenko Y. , A Comprehensive Approach to the Assessment and Management of Posttraumatic Headache, Physical Medicine and Rehabilitation Clinics of North America. (2025) 36, no. 4, 749–761, 10.1016/j.pmr.2025.07.003. 41167854

[8] Orr T. J. , Lesha E. , Kramer A. H. et al., Traumatic Brain Injury: a Comprehensive Review of Biomechanics and Molecular Pathophysiology, World Neurosurgery. (2024) 185, 74–88, 10.1016/j.wneu.2024.01.084. 38272305

[9] Simon D. W. , McGeachy M. J. , Bayır H. , Clark R. S. B. , Loane D. J. , and Kochanek P. M. , The far-reaching Scope of Neuroinflammation After Traumatic Brain Injury, Nature Reviews Neurology. (2017) 13, no. 3, 171–191, 10.1038/nrneurol.2017.13. 28186177 PMC5675525

[10] Sahbaie P. , Irvine K. A. , Shi Xy , and Clark J. D. , Monoamine Control of Descending Pain Modulation After Mild Traumatic Brain Injury, Scientific Reports. (2022) 12, no. 1, 10.1038/s41598-022-20292-7. PMC9522857 36175479

[11] Loane D. J. and Kumar A. , Microglia in the TBI Brain: the Good, the Bad, and the Dysregulated, Experimental Neurology. (2016) 275, no. 0 3, 316–327, 10.1016/j.expneurol.2015.08.018. 26342753 PMC4689601

[12] Donat C. K. , Scott G. , Gentleman S. M. , and Sastre M. , Microglial Activation in Traumatic Brain Injury, Frontiers in Aging Neuroscience. (2017) 9, 10.3389/fnagi.2017.00208. PMC5487478 28701948

[13] Nagarajan G. and Zhang Y. , Distinct Expression Profile Reveals Glia Involvement in the Trigeminal System Attributing to Post-Traumatic Headache, The Journal of Headache and Pain. (2024) 25, no. 1, 10.1186/s10194-024-01897-x. PMC11585153 39578726

[14] Smajkan A. , Naranjo-Cinto E. , and Bareyre F. M. , Mild and Repetitive Mild Traumatic Brain Injury: Changes in Microglial Cells and Synapses, Neural Regeneration Research. (2026) 21, no. 10, 4578–4593, 10.4103/nrr.nrr-d-25-00902. 41467392 PMC13568626

[15] Witcher K. G. , Bray C. E. , Chunchai T. et al., Traumatic Brain Injury Causes Chronic Cortical Inflammation and Neuronal Dysfunction Mediated by Microglia, Journal of Neuroscience: The Official Journal of the Society for Neuroscience. (2021) 41, no. 7, 1597–1616, 10.1523/jneurosci.2469-20.2020. 33452227 PMC7896020

[16] Koupourtidou C. , Schwarz V. , Aliee H. et al., Shared Inflammatory Glial Cell Signature After Stab Wound Injury, Revealed by Spatial, Temporal, and Cell-Type-Specific Profiling of the Murine Cerebral Cortex, Nature Communications. (2024) 15, no. 1, 10.1038/s41467-024-46625-w. PMC10991294 38570482

[17] Zhang H. , Zhang X. , Chai Y. , Wang Y. , Zhang J. , and Chen X. , Astrocyte-Mediated Inflammatory Responses in Traumatic Brain Injury: Mechanisms and Potential Interventions, Frontiers in Immunology. (2025) 16, 10.3389/fimmu.2025.1584577. PMC12094960 40406119

[18] Paolicelli R. C. , Sierra A. , Stevens B. et al., Microglia States and Nomenclature: A Field at its Crossroads, Neuron. (2022) 110, no. 21, 3458–3483, 10.1016/j.neuron.2022.10.020. 36327895 PMC9999291

[19] Keren-Shaul H. , Spinrad A. , Weiner A. et al., A Unique Microglia Type Associated With Restricting Development of Alzheimer′s Disease, Cell. (2017) 169, no. 7, 1276–1290.e17, 10.1016/j.cell.2017.05.018. 28602351

[20] Mathys H. , Adaikkan C. , Gao F. et al., Temporal Tracking of Microglia Activation in Neurodegeneration at Single-Cell Resolution, Cell Reports. (2017) 21, no. 2, 366–380, 10.1016/j.celrep.2017.09.039. 29020624 PMC5642107

[21] Hammond T. R. , Dufort C. , Dissing-Olesen L. et al., Single-Cell RNA Sequencing of Microglia Throughout the Mouse Lifespan and in the Injured Brain Reveals Complex Cell-State Changes, Immunity. (2019) 50, no. 1, 253–271.e6, 10.1016/j.immuni.2018.11.004. 30471926 PMC6655561

[22] Gottlieb A. , Toledano-Furman N. , Prabhakara K. S. et al., Time Dependent Analysis of Rat Microglial Surface Markers in Traumatic Brain Injury Reveals Dynamics of Distinct Cell Subpopulations, Scientific Reports. (2022) 12, no. 1, 10.1038/s41598-022-10419-1. PMC9012748 35428862

[23] Izzy S. , Liu Q. , Fang Z. et al., Time-Dependent Changes in Microglia Transcriptional Networks Following Traumatic Brain Injury, Frontiers in Cellular Neuroscience. (2019) 13, 10.3389/fncel.2019.00307. PMC6694299 31440141

[24] Nie W. , Yan J. , Xie Z. et al., Single-Cell Omics Analysis Reveals Critical Cell Subtypes and Their Functional Attributes in Myocardial Ischemia-Reperfusion Injury, BIO Integration. (2026) 7, no. 1, 10.15212/bioi-2025-0159.

[25] Zhao Z. , Cai H. , Zhao Z. et al., Cancer-Associated Fibroblast-Derived GDF15 Induces Oxidative Stress and Neutrophil Infiltration in Head and Neck Squamous Cell Carcinoma Through the PI3K/AKT/STAT3 Axis Cascade, Research: Ideas for Today′s Investors, Washington, DC. (2025) 8, 10.34133/research.0901. PMC12480759 41035818

[26] Zhao Z. , Zhao Z. , Lin Z. et al., Decoding Multiple Myeloma: Single-Cell Insights into Tumor Heterogeneity, Immune Dynamics, and Disease Progression, Frontiers in Immunology. (2025) 16, 10.3389/fimmu.2025.1584350. PMC12095158 40406148

[27] Stuart T. , Butler A. , Hoffman P. et al., Comprehensive Integration of Single-Cell Data, Cell. (2019) 177, no. 7, 1888–1902.e21, 10.1016/j.cell.2019.05.031. 31178118 PMC6687398

[28] Zhao Z. , Cai H. , Nie W. et al., Ectopic Expression of GDF15 in Cancer-Associated Fibroblasts Enhances Melanoma Immunosuppression via the GFRAL/RET Cascade, Journal for ImmunoTherapy of Cancer. (2025) 13, no. 6, 10.1136/jitc-2024-011036. PMC12198796 40555562

[29] Zhao Z. , Dong Y. , Zhao Z. , Xiahou Z. , and Sun C. , Single-Cell Atlas of Endothelial Cells in Atherosclerosis: Identifying C1 CXCL12+ Ecs as Key Proliferative Drivers for Immunological Precision Therapeutics in Atherosclerosis, Frontiers in Immunology. (2025) 16, 10.3389/fimmu.2025.1569988. PMC12104226 40421026

[30] McGinnis C. S. , Murrow L. M. , and Gartner Z. J. , Doubletfinder: Doublet Detection in Single-Cell RNA Sequencing Data Using Artificial Nearest Neighbors, Cell Systems. (2019) 8, no. 4, 329–337.e4, 10.1016/j.cels.2019.03.003. 30954475 PMC6853612

[31] Lin L. , Zou J. , Pei S. et al., Germinal Center B-cell Subgroups in the Tumor Microenvironment Cannot be Overlooked: Their Involvement in Prognosis, Immunotherapy Response, and Treatment Resistance in Head and Neck Squamous Carcinoma, Heliyon. (2024) 10, no. 19, 10.1016/j.heliyon.2024.e37726. PMC11466559 39391510

[32] Ge Q. , Zhao Z. , Li X. et al., Deciphering the Suppressive Immune Microenvironment of Prostate Cancer Based on CD4+ Regulatory T Cells: Implications for Prognosis and Therapy Prediction, Clinical and Translational Medicine. (2024) 14, no. 1, 10.1002/ctm2.1552. PMC10797244 38239097

[33] Korsunsky I. , Millard N. , Fan J. et al., Fast, Sensitive and Accurate Integration of single-cell Data with Harmony, Nature Methods. (2019) 16, no. 12, 1289–1296, 10.1038/s41592-019-0619-0. 31740819 PMC6884693

[34] Traynor S. , Jakobsen M. K. , Green T. M. et al., Single-Cell Sequencing Unveils Extensive Intratumoral Heterogeneity of Cancer/Testis Antigen Expression in Melanoma and Lung Cancer, Journal for ImmunoTherapy of Cancer. (2024) 12, no. 6, 10.1136/jitc-2023-008759. PMC11184195 38886115

[35] Wang Y. , Zheng M. , Zhang Y. et al., Single‐Cell Transcriptomic Analysis of the Immune Response to COVID‐19 and Tuberculosis Coinfection, Exploration. (2025) 8. 10.1002/EXP.20240022 PMC12561472 41163794

[36] Jin X. , Jian Z. , Ma Y. et al., Single-Cell RNA Sequencing Analysis Reveals the Role of Macrophage-Mediated CD44–AKT–CCL2 Pathways in Renal Tubule Injury During Calcium Oxalate Crystal Formation, Research: Ideas for Today′s Investors. (2025) 8, 10.34133/research.0690. PMC12053376 40330661

[37] Shi Y. , Wu Y. , Li F. et al., Investigating the Immunogenic Cell Death-dependent Subtypes and Prognostic Signature of Triple-Negative Breast Cancer, Phenomics. (2024) 4, no. 1, 34–45, 10.1007/s43657-023-00133-x. 38605910 PMC11003942

[38] Peng Y. , Xiong L. B. , Zhou Z. H. et al., Single-Cell Transcriptomics Reveals a Low CD8(+) T Cell Infiltrating State Mediated by Fibroblasts in Recurrent Renal Cell Carcinoma, Journal for ImmunoTherapy of Cancer. (2022) 10, no. 2, 10.1136/jitc-2021-004206. PMC8819783 35121646

[39] Wang X. , Li J. , and Ning J. , Comprehensive Multi‐Omics Integration Reveals B Cell‐Derived ELL2 as a Novel Diagnostic and Prognostic Biomarker in Sepsis, Media Research. (2025) 1, no. 2, 170–174, 10.1002/mdr2.70010.

[40] Feng X. , Luo Z. , Zhang W. et al., Zn‐Dhm Nanozymes Enhance Muscle Regeneration Through ROS Scavenging and Macrophage Polarization in Volumetric Muscle Loss Revealed by Single‐Cell Profiling, Advanced Functional Materials. (2025) 35, no. 35, 10.1002/adfm.202506476.

[41] Tong Z. , Mang G. , Wang D. , Cui J. , Yang Q. , and Zhang M. , Single-Cell RNA Sequencing Maps Immune Cell Heterogeneity in Mice With Allogeneic Cardiac Transplantation, Cardiovascular Innovations and Applications. (2023) 8, no. 1, 10.15212/cvia.2023.0023.

[42] Gulati G. S. , Sikandar S. S. , Wesche D. J. et al., Single-Cell Transcriptional Diversity is a Hallmark of Developmental Potential, Science (New York, N.Y.). (2020) 367, no. 6476, 405–411, 10.1126/science.aax0249. 31974247 PMC7694873

[43] Street K. , Risso D. , Fletcher R. B. et al., Slingshot: Cell Lineage and Pseudotime Inference for Single-Cell Transcriptomics, BMC Genomics. (2018) 19, no. 1, 10.1186/s12864-018-4772-0. PMC6007078 29914354

[44] Yang X. , Huang M. , Chen H. et al., Biomaterial-Mediated Cell Atlas: An Insight From Single-Cell and Spatial Transcriptomics, Bioactive Materials. (2025) 54, 1–33, 10.1016/j.bioactmat.2025.07.047. 40821937 PMC12356410

[45] Jin S. , Guerrero-Juarez C. F. , Zhang L. et al., Inference and Analysis of Cell-Cell Communication Using Cellchat, Nature Communications. (2021) 12, no. 1, 10.1038/s41467-021-21246-9. PMC7889871 33597522

[46] Su D. , Jiao Z. , Li S. et al., Spatiotemporal Single-Cell Transcriptomic Profiling Reveals Inflammatory Cell States in a Mouse Model of Diffuse Alveolar Damage, Exploration. (2023) 3, no. 3, 10.1002/exp.20220171. PMC10624389 37933384

[47] Wu J. , Hu N. , Guo B. et al., Single-Cell Transcriptomics Systematically Discloses the Immune-Inflammatory Responses of Peripheral Blood Mononuclear Cells for Diabetic Nephropathy, Genes & Diseases. (2023) 11, no. 4, 10.1016/j.gendis.2023.06.007. PMC10951445 38510479

[48] Ouyang Y. , Hong Y. , Mai C. et al., Transcriptome Analysis Reveals Therapeutic Potential of NAMPT in Protecting Against Abdominal Aortic Aneurysm in Human and Mouse, Bioactive Materials. (2023) 34, 17–36, 10.1016/j.bioactmat.2023.11.020. 38173843 PMC10761368

[49] Aibar S. , González-Blas C. B. , Moerman T. et al., SCENIC: Single-Cell Regulatory Network Inference and Clustering, Nature Methods. (2017) 14, no. 11, 1083–1086, 10.1038/nmeth.4463. 28991892 PMC5937676

[50] Irvine K. and Clark J. D. , Chronic Pain After Traumatic Brain Injury: Pathophysiology and Pain Mechanisms, Pain Medicine. (2018) 19, no. 7, 1315–1333, 10.1093/pm/pnx153. 29025157

[51] Da Silva Fiorin F. , do Espírito Santo C. C. , Da Silva J. T. , and Chung M. K. , Inflammation, Brain Connectivity, and Neuromodulation in Post-traumatic Headache, Brain, behavior, & immunity- health. (2024) 35, 10.1016/j.bbih.2024.100723. PMC10827408 38292321

[52] Jassam Y. N. , Izzy S. , Whalen M. , McGavern D. B. , and El Khoury J. , Neuroimmunology of Traumatic Brain Injury: Time for a Paradigm Shift, Neuron. (2017) 95, no. 6, 1246–1265, 10.1016/j.neuron.2017.07.010. 28910616 PMC5678753

[53] Todd B. P. , Chimenti M. S. , Luo Z. , Ferguson P. J. , Bassuk A. G. , and Newell E. A. , Traumatic Brain Injury Results in Unique Microglial and Astrocyte Transcriptomes Enriched for Type I Interferon Response, Journal of Neuroinflammation. (2021) 18, no. 1, 10.1186/s12974-021-02197-w. PMC8259035 34225752

[54] Burda J. E. , Bernstein A. M. , and Sofroniew M. V. , Astrocyte Roles in Traumatic Brain Injury, Experimental Neurology. (2016) 275, no. 0 3, 305–315, 10.1016/j.expneurol.2015.03.020. 25828533 PMC4586307

[55] Armstrong R. C. , Mierzwa A. J. , Sullivan G. M. , and Sanchez M. A. , Myelin and Oligodendrocyte Lineage Cells in White Matter Pathology and Plasticity After Traumatic Brain Injury, Neuropharmacology. (2016) 110, no. Pt B, 654–659, 10.1016/j.neuropharm.2015.04.029. 25963414

[56] Salter M. W. and Stevens B. , Microglia Emerge as Central Players in Brain Disease, Nature Medicine. (2017) 23, no. 9, 1018–1027, 10.1038/nm.4397. 28886007

[57] Butovsky O. , Jedrychowski M. P. , Moore C. S. et al., Identification of a Unique TGF-β-dependent Molecular and Functional Signature in Microglia, Nature Neuroscience. (2014) 17, no. 1, 131–143, 10.1038/nn.3599. 24316888 PMC4066672

[58] Paglinawan R. , Malipiero U. , Schlapbach R. , Frei K. , Reith W. , and Fontana A. , Tgfbeta Directs Gene Expression of Activated Microglia to an Anti-inflammatory Phenotype Strongly Focusing on Chemokine Genes and Cell Migratory Genes, Glia. (2003) 44, no. 3, 219–231, 10.1002/glia.10286. 14603463

[59] Caraci F. , Gulisano W. , Guida C. A. et al., A Key Role for TGF-β1 in Hippocampal Synaptic Plasticity and Memory, Scientific Reports. (2015) 5, no. 1, 10.1038/srep11252. PMC4462026 26059637

[60] Lalive P. H. , Paglinawan R. , Biollaz G. et al., TGF-beta-treated Microglia Induce Oligodendrocyte Precursor Cell Chemotaxis Through the HGF-c-Met Pathway, European Journal of Immunology. (2005) 35, no. 3, 727–737, 10.1002/eji.200425430. 15724248

[61] Kawasaki Y. , Kohno T. , Zhuang Z. Y. et al., Ionotropic and Metabotropic Receptors, Protein Kinase A, Protein Kinase C, and Src Contribute to C-fiber-induced ERK Activation and Camp Response element-binding Protein Phosphorylation in Dorsal Horn Neurons, Leading to Central Sensitization, Journal of Neuroscience: The Official Journal of the Society for Neuroscience. (2004) 24, no. 38, 8310–8321, 10.1523/jneurosci.2396-04.2004. 15385614 PMC6729681

[62] Svensson C. I. , Marsala M. , Westerlund A. et al., Activation of p38 Mitogen-Activated Protein Kinase in Spinal Microglia is a Critical Link in Inflammation-Induced Spinal Pain Processing, Journal of Neurochemistry. (2003) 86, no. 6, 1534–1544, 10.1046/j.1471-4159.2003.01969.x. 12950462

[63] Ji R. , Nackley A. , Huh Y. , Terrando N. , and Maixner W. , Neuroinflammation and Central Sensitization in Chronic and Widespread Pain, Anesthesiology. (2018) 129, no. 2, 343–366, 10.1097/aln.0000000000002130. 29462012 PMC6051899

[64] Kuner R. and Flor H. , Structural Plasticity and Reorganisation in Chronic Pain, Nature Reviews Neuroscience. (2016) 18, no. 1, 20–30, 10.1038/nrn.2016.162. 27974843

[65] Schafer D. P. , Lehrman E. , Kautzman A. et al., Microglia Sculpt Postnatal Neural Circuits in an Activity and Complement-Dependent Manner, Neuron. (2012) 74, no. 4, 691–705, 10.1016/j.neuron.2012.03.026. 22632727 PMC3528177

[66] Inoue K. and Tsuda M. , Microglia in Neuropathic Pain: Cellular and Molecular Mechanisms and Therapeutic Potential, Nature Reviews Neuroscience. (2018) 19, no. 3, 138–152, 10.1038/nrn.2018.2. 29416128

[67] Zhang Y. , Wang T. , Wu S. et al., Notch Signaling Pathway: A New Target for Neuropathic Pain Therapy, The Journal of Headache and Pain. (2023) 24, no. 1, 10.1186/s10194-023-01616-y. PMC10349482 37454050

[68] Tang Y. , Chen Y. , Liu R. , Li W. , Hua B. , and Bao Y. , Wnt Signaling Pathways: A Role in Pain Processing, Neuromolecular Medicine. (2022) 24, no. 3, 233–249, 10.1007/s12017-021-08700-z. 35067780 PMC9402773

[69] Cornell J. , Salinas S. , Huang H. Y. et al., Microglia Regulation of Synaptic Plasticity and Learning and Memory, Neural Regeneration Research. (2022) 17, no. 4, 705–716. 34472455 10.4103/1673-5374.322423 PMC8530121

[70] Pawelec P. , Ziemka-Nalecz M. , Sypecka J. , and Zalewska T. , The Impact of the CX3CL1/CX3CR1 Axis in Neurological Disorders, Cells. (2020) 9, no. 10, 10.3390/cells9102277. PMC7600611 33065974

[71] Febinger H. Y. , Thomasy H. E. , Pavlova M. N. et al., Time-Dependent Effects of CX3CR1 in a Mouse Model of Mild Traumatic Brain Injury, Journal of Neuroinflammation. (2015) 12, no. 1, 10.1186/s12974-015-0386-5. PMC4557842 26329692

[72] Camacho-Hernández N. P. and Peña-Ortega F. , Fractalkine/CX3CR1-Dependent Modulation of Synaptic and Network Plasticity in Health and Disease, Neural Plasticity. (2023) 2023, 4637073–17, 10.1155/2023/4637073. 36644710 PMC9833910

[73] Silva R. and Malcangio M. , Fractalkine/CX(3)CR(1) Pathway in Neuropathic Pain: An Update, Frontiers in pain research (Lausanne, Switzerland). (2021) 2, 10.3389/fpain.2021.684684. PMC8915718 35295489

[74] Krieger T. G. , Moran C. M. , Frangini A. et al., Mutations in Thyroid Hormone Receptor Α1 Cause Premature Neurogenesis and Progenitor Cell Depletion in Human Cortical Development, Proceedings of the National Academy of Sciences of the United States of America. (2019) 116, no. 45, 22754–22763, 10.1073/pnas.1908762116. 31628250 PMC6842615

[75] Bernal J. , Thyroid Hormone Receptors in Brain Development and Function. Nature Clinical Practice, Endocrinology and Metabolism. (2007) 3, no. 3, 249–259, 10.1038/ncpendmet0424. 17315033

[76] Lima F. R. , Gervais A. , Colin C. , Izembart M. , Neto V. M. , and Mallat M. , Regulation of Microglial Development: A Novel Role for Thyroid Hormone, Journal of Neuroscience: The Official Journal of the Society for Neuroscience. (2001) 21, no. 6, 2028–2038, 10.1523/jneurosci.21-06-02028.2001. 11245686 PMC6762591

[77] Mori Y. , Tomonaga D. , Kalashnikova A. et al., Effects of 3,3′,5-Triiodothyronine on Microglial Functions, Glia. (2015) 63, no. 5, 906–920, 10.1002/glia.22792. 25643925

[78] Ziegler K. , Folkard R. , Gonzalez A. J. et al., Primary Somatosensory Cortex Bidirectionally Modulates Sensory Gain and Nociceptive Behavior in a Layer-Specific Manner, Nature Communications. (2023) 14, no. 1, 10.1038/s41467-023-38798-7. PMC10209111 37225702

[79] Krukowski K. , Nolan A. , Becker M. et al., Novel microglia-mediated Mechanisms Underlying Synaptic Loss and Cognitive Impairment After Traumatic Brain Injury, Brain, Behavior, and Immunity. (2021) 98, 122–135, 10.1016/j.bbi.2021.08.210. 34403733 PMC9119574

[80] Yi M. , Liu Y. , Liu Y. U. et al., Optogenetic Activation of Cortical Microglia Promotes Neuronal Activity and Pain Hypersensitivity, Cell Reports. (2025) 44, no. 5, 10.1016/j.celrep.2025.115717. PMC12161772 40381194

[81] Fülöp B. , Hunyady Á. , Bencze N. et al., IL-1 Mediates Chronic Stress-Induced Hyperalgesia Accompanied by Microglia and Astroglia Morphological Changes in Pain-Related Brain Regions in Mice, International Journal of Molecular Sciences. (2023) 24, no. 6, 10.3390/ijms24065479. PMC10052634 36982563

[82] Gu N. , Yi M. H. , Murugan M. et al., Spinal Microglia Contribute to Sustained Inflammatory Pain via Amplifying Neuronal Activity, Molecular Brain. (2022) 15, no. 1, 10.1186/s13041-022-00970-3. PMC9609165 36289499

[83] Taylor A. M. W. , Mehrabani S. , Liu S. , Taylor A. J. , and Cahill C. M. , Topography of Microglial Activation in Sensory- and Affect-Related Brain Regions in Chronic Pain, Journal of Neuroscience Research. (2017) 95, no. 6, 1330–1335, 10.1002/jnr.23883. 27574286 PMC5332533

